# High-Throughput Profiling of Candida auris Isolates Reveals Clade-Specific Metabolic Differences

**DOI:** 10.1128/spectrum.00498-23

**Published:** 2023-04-25

**Authors:** Philipp Brandt, Mohammad H. Mirhakkak, Lysett Wagner, Dominik Driesch, Anna Möslinger, Pauline Fänder, Sascha Schäuble, Gianni Panagiotou, Slavena Vylkova

**Affiliations:** a Septomics Research Center, Friedrich Schiller University, and Leibniz Institute for Natural Product Research and Infection Biology, Hans Knöll Institute, Jena, Germany; b Systems Biology and Bioinformatics, Leibniz Institute for Natural Product Research and Infection Biology, Hans Knöll Institute, Jena, Germany; c BioControl Jena, Jena, Germany; d Microbial Pathogenicity Mechanisms, Leibniz Institute for Natural Product Research and Infection Biology, Hans Knöll Institute, Jena, Germany; University of Guelph

**Keywords:** *Candida auris*, dipeptide transport, carboxylic acids, phenotypic profiling, metabolism, Jen2, dicarboxylic acids, dipeptides

## Abstract

Candida auris, a multidrug-resistant human fungal pathogen that causes outbreaks of invasive infections, emerged as four distinct geographical clades. Previous studies identified genomic and proteomic differences in nutrient utilization on comparison to Candida albicans, suggesting that certain metabolic features may contribute to C. auris emergence. Since no high-throughput clade-specific metabolic characterization has been described yet, we performed a phenotypic screening of C. auris strains from all 4 clades on 664 nutrients, 120 chemicals, and 24 stressors. We identified common and clade- or strain-specific responses, including the preferred utilization of various dipeptides as nitrogen source and the inability of the clade II isolate AR 0381 to withstand chemical stress. Further analysis of the metabolic properties of C. auris isolates showed robust growth on intermediates of the tricarboxylic acid cycle, such as citrate and succinic and malic acids. However, there was reduced or no growth on pyruvate, lactic acid, or acetate, likely due to the lack of the monocarboxylic acid transporter Jen1, which is conserved in most pathogenic *Candida* species. Comparison of C. auris and C. albicans transcriptomes of cells grown on alternative carbon sources and dipeptides as a nitrogen source revealed common as well as species-unique responses. C. auris induced a significant number of genes with no ortholog in C. albicans, e.g., genes similar to the nicotinic acid transporter *TNA1* (alternative carbon sources) and to the oligopeptide transporter (*OPT*) family (dipeptides). Thus, C. auris possesses unique metabolic features which could have contributed to its emergence as a pathogen.

**IMPORTANCE** Four main clades of the emerging, multidrug-resistant human pathogen Candida auris have been identified, and they differ in their susceptibilities to antifungals and disinfectants. Moreover, clade- and strain-specific metabolic differences have been identified, but a comprehensive overview of nutritional characteristics and resistance to various stressors is missing. Here, we performed high-throughput phenotypic characterization of C. auris on various nutrients, stressors, and chemicals and obtained transcriptomes of cells grown on selected nutrients. The generated data sets identified multiple clade- and strain-specific phenotypes and induction of C. auris-specific metabolic genes, showing unique metabolic properties. The presented work provides a large amount of information for further investigations that could explain the role of metabolism in emergence and pathogenicity of this multidrug-resistant fungus.

## INTRODUCTION

Candida auris is a human fungal pathogen that poses a major concern to health care systems worldwide, as it has been associated with major outbreaks of invasive infections at various geographical locations. Its high resistance to multiple antifungals and common disinfectants, as well as its persistent colonization on human skin, facilitates patient-to-patient transmission, which makes the containment of hospital outbreaks extremely challenging (1, 2). Genome sequencing analyses of clinical isolates defined the existence of four distinct C. auris clades that grouped dependent on their geographical location: South Asian (clade I), East Asian (clade II), African (clade III), and South American (clade IV) (3, 4). Recently, an isolate recovered from a patient in Iran suggested the existence of a fifth clade (clade V), and this requires further validation (5). While the clades differ by tens of thousands of single-nucleotide polymorphisms, the strains within a clade are highly related and therefore almost clonal (6). This suggests that C. auris emerged independently and simultaneously in at least four geographic locations, making the infection potential of this agent even more alarming.

Antifungal susceptibility studies revealed that 90% of all C. auris isolates are resistant to fluconazole, 8% to amphotericin B, and 2% to echinocandins (7). Comparison of the genomes of multiple C. auris isolates to those of other *Candida* species indicated a notable expansion of genes linked to drug resistance, virulence, and nutrient transport (4). Specifically, this and other studies revealed mutations and high-copy-number variants in *ERG11*, encoding the fluconazole target lanosterol 14-α-demethylase, and overexpression of efflux pumps, like Cdr1 (4, 7, 8). Alarmingly, serial exposure of C. auris to azoles, polyenes, and echinocandins resulted in the emergence of 10 nonsynonymous mutations in 8 genes, that evolved in 5 separate evolutionary lineages, including novel mutations (9). Moreover, treatment with antifungal drugs enhanced the expression of known drug resistance genes and of several either unique or expanded gene families, including iron and oligopeptide transporters, secreted lipases, and metabolic regulators previously linked to drug resistance in the well-studied pathogen Candida albicans (4). While the clade-specific differences in antifungal resistance have been explored in detail, little is known about C. auris virulence and the clade-specific phenotypic characteristics. Detailed phenotypic characterization of C. auris metabolic features is available only for a clade II isolate (type strain JCM 15448), which possesses overall lower antifungal resistance than the other clades (10, 11). Moreover, in contrast to clade II, clade I isolates are known to assimilate *N*-acetylglucosamine (GlcNAc), which is potentially linked to the increased antifungal resistance of strains from this clade (10, 12).

The aim of this study was to obtain an extensive phenotypic portrait of C. auris isolates from the four main clades and to focus on the clade-specific characteristics, which may aid species identification and explain the rapid emergence as a major pathogen. Therefore, we utilized phenotype microarrays for microbial cells to perform a high-throughput phenotypic profiling of 10 C. auris isolates representing the four main clades. We measured the metabolic activity on 664 nutrients, 24 stressors, and 120 chemicals, including commonly used antifungals, and we identified 6 chemicals that inhibited the growth of all C. auris isolates. Furthermore, all C. auris isolates were able to utilize many different nitrogen sources, including various dipeptides. Growth on various monocarboxylic acids as carbon source, on the other hand, was either poor or absent, whereas di- and tricarboxylic acids supported overall robust growth of C. auris. Subsequent transcriptomic profiling of the C. auris clade I reference strain AR 0389 and Candida albicans reference strain SC5314 revealed a C. auris-specific transcriptional response to alternative carbon sources and dipeptides as nitrogen source. Altogether, this study identified strain-, clade-, and species-specific metabolic characteristics in C. auris which, together with other factors, could have contributed to its emergence as a major human fungal pathogen.

## RESULTS

### Carbon sources commonly associated with the human host were well utilized by all C. auris clades.

In order to identify C. auris clade-specific phenotypic signatures, we performed a high-throughput phenotypic screen utilizing the phenotype microarrays for microbial cells (Biolog, Hayward, CA, USA). We tested the metabolic activity on 664 nutrients, 120 chemicals, and 24 stressors for 10 C. auris isolates from clades I to IV in triplicates. The analyzed data sets in Fig. S1 and Table S2 in the supplemental material show the following for each isolate and condition: (i) exponential growth (purple, growth above the threshold, value of 1) or nonexponential growth (yellow, no growth or growth below the threshold, value of 0) growth; (ii) total growth over time (0 to 24 h or 0 to 48 h) based on the area under the curve (AUC). We also calculated the AUC ratio of the median value from all isolates per clade for each microarray plate to gain insight into the clade-specific differences in metabolic activity (Table S2).

**FIG 1:**
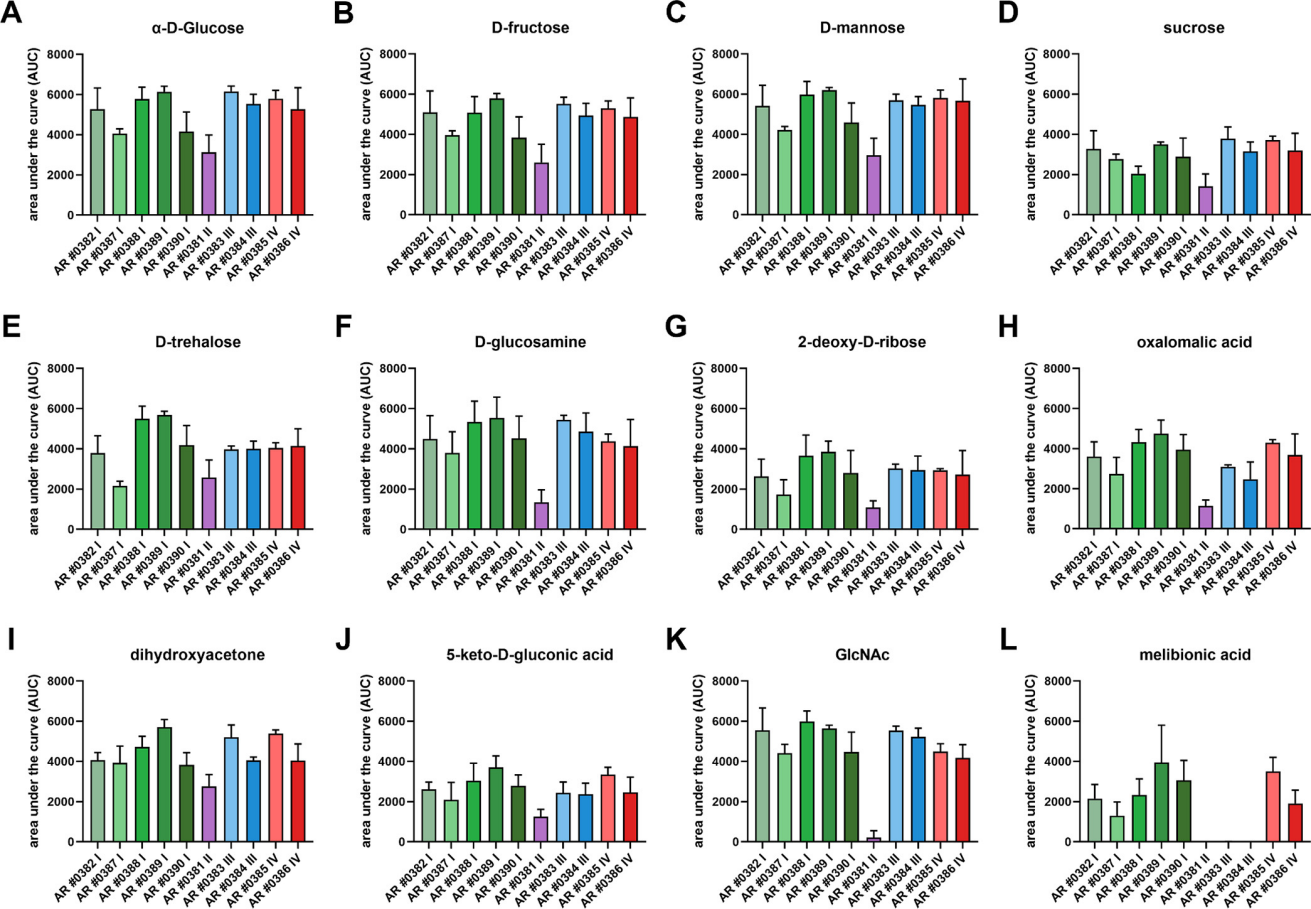
Phenotypic screen of C. auris isolates on various carbon sources. Biolog Phenotypic MicroArray plates PM1 and PM2 (carbon sources) for fungi were used to measure the metabolic activity of C. auris isolates kinetically every 15 min for 48 h at 37°C. The bars represent the calculated means and standard deviations of the AUC of selected carbon sources of three biological replicates per strain.

The ability to utilize various host-associated carbon sources provides an advantage in fungal survival and pathogenicity. The phenotype microarray screen included 190 carbon sources (PM1, PM2), 10 of which supported exponential growth of each C. auris isolate (Fig. 1A to J; Fig. S1A and B). These included various monosaccharides (dihydroxyacetone, α-d-glucose, d-fructose, d-mannose, 2-deoxy-d-ribose), disaccharides (sucrose, d-trehalose), amino sugar (d-glucosamine), and organic acids (5-keto-d-gluconic acid, oxalomalic acid) (Fig. 1A to J; Fig. S1A and B). According to the AUC values, reflecting growth over time, the most preferred carbon sources of all clades were d-mannose and α-d-glucose (Fig. S1A, Table S2). The isolate from clade II generally had the lowest capability to utilize different carbon sources, as exponential growth was detected only on 16 substrates (Fig. S1A and B, Table S2). For example, this isolate exhibited no exponential growth on d-arabinose, maltose, maltotriose, melibionic acid, d-melezitose, GlcNAc, palatinose, pectin, or turanose (Fig. S1A and B). All isolates from clades I, III, and IV showed high metabolic activity on GlcNAc, while the isolate from clade II (AR 0381) failed to grow on this carbon source (Fig. 1K). Other clade-specific growth characteristics were also noted, including lack of exponential growth of clade III on d-arabinose, d-ribose, and melibionic acid and of clade IV on pectin and d-melezitose (Fig. 1L; Fig. S1A and B). Only the two isolates from clade III (AR 0383 and AR 0384) and one isolate from clade I (AR 0390) grew to exponential phase on laminarin, a polysaccharide found in algae (Fig. S1B). We observed that only clade IV isolates utilized the monosaccharide d-tagatose, a sweetener used in the food industry (Fig. S1B).

Thus, mannose and glucose are the preferred carbon sources for all four C. auris clades, while several isolates of different clades showed variable growth characteristics on different carbon sources.

### C. auris grew exceptionally well on various dipeptides as a nitrogen source.

The phenotype microarrays for microbial cells allow testing for fungal growth on 380 nitrogen sources (PM3 and PM6 to -8). Within this panel were 95 nitrogen sources that included all proteinogenic amino acids, amines, amino sugars, and inorganic nitrogen sources and 285 peptide nitrogen sources, such as various di- and tripeptides. C. auris isolates from clade I exhibited exponential growth on most different nitrogen sources compared to the other clades. In addition, all clade I isolates and the isolate of clade II AR 0381 were the most versatile utilizing dipeptide nitrogen sources, whereas the clade III isolates were the least versatile (Fig. S1E to G, Table S2). The most preferred nitrogen sources for all isolates were l-glutamine, His-, Ala-, or Gln-containing dipeptides, and ammonia, as growth on these nutrients yielded the highest AUC values (Table S2). The isolates from clades II and III showed no exponential growth on GlcNAc as nitrogen source, while only clade II failed to utilize this nutrient as a carbon source (Fig. S1C, Table S2).

Interestingly, while all isolates were able to utilize l-alanine, l-arginine, l-asparagine, l-aspartic acid, l-glutamic acid, l-glutamine, glycine, and l-serine as nitrogen source, only isolates from clade I were able to utilize l-valine, l-isoleucine, l-lysine, l-leucine, l-phenylalanine, and l-tryptophan (Fig. S1C, Table S2). This was also reflected in their increased ability to use dipeptides that contained these amino acids, especially with the aromatic amino acid-containing dipeptides. Notable, the isolates from clade I could be divided into two groups regarding their ability to utilize nitrogen peptides: isolates AR 0388 and AR 0389 exhibited exponential growth on fewer dipeptides (102 and 104, respectively) with a clear preference toward His-containing dipeptides. The other isolates showed exponential growth on 154 (AR 0382), 186 (AR 0387), and 165 (AR 0390) different dipeptide combinations (Fig. S1E to G). The isolate from clade II also utilized nitrogen sources quite well, since it grew on 137 peptide nitrogen sources with a preference toward Ala-containing dipeptides. The isolates from clades III and IV exhibited exponential growth mostly on His-containing peptides (Fig. S1E to G, Table S2). In summary, C. auris grows exceptionally well on various nitrogen peptide sources. All clades preferred His-containing dipeptides except for the isolate from clade II, which preferred Ala-containing dipeptides.

### C. auris showed strain-specific utilization of sulfur sources.

**FIG 2:**
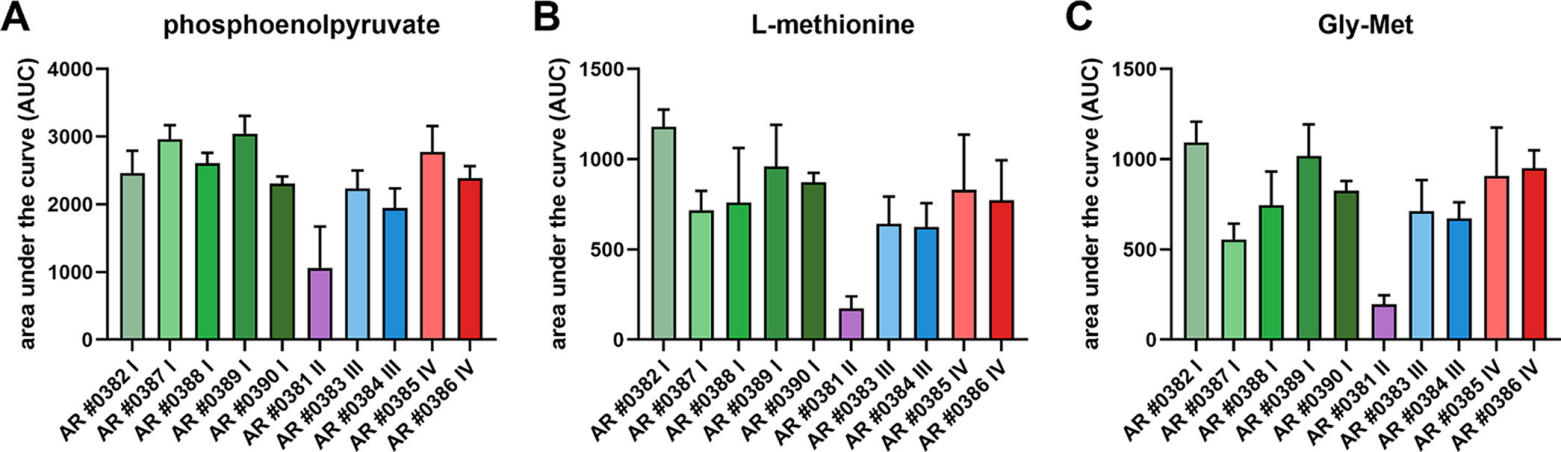
Phenotypic screen of C. auris isolates on various phosphorus and sulfur sources. Biolog Phenotypic MicroArray plate PM4 (phosphorus and sulfur sources) for fungi was used to measure the metabolic activity of C. auris isolates kinetically every 15 min for 48 h at 37°C. The bars represent the calculated means and standard deviations of the AUC of three biological replicates per strain grown on phosphoenolpyruvate (A), l-methionine (B), or Gly-Met (C).

C. auris utilized a variety of phosphorus sources, as all isolates reached the exponential phase on 40 of 59 tested phosphorus sources (Fig. S1D). One of the most preferred phosphorus sources for all isolates was phosphoenolpyruvate, an intermediate of glycolysis and gluconeogenesis (Fig. 2A). Of the 35 tested sulfur sources, only 10 supported exponential growth in all isolates, and 16 supported growth in at least one isolate. Most of the C. auris isolates preferred methionine-containing sulfur sources (l-methionine, l-methionine sulfoxide, Gly-Met, or N-acetyl-d,l-methionine) (Fig. 2B and C; Fig. S1D). Thus, all C. auris isolates utilized the same phosphorus sources, whereas the utilization of sulfur sources was more strain specific.

### Isolates from clade I and III were less susceptible to osmotic stress than isolates from clade II and IV.

In consensus with others (13, 14), all C. auris isolates were able to tolerate high salt concentrations, such as 100 mM ammonium sulfate, 200 mM sodium phosphate, or 100 mM sodium nitrate (Fig. S1H, Table S2). However, we noted that isolates from clades I and III were more resistant to osmotic stressors than were isolates from clades II and IV. For instance, clades I and III isolates tolerated high concentrations of sodium chloride (up to 8% for individual isolates), whereas isolates from clades II and IV showed no exponential growth at concentrations higher than 3% (Fig. S1H). Similarly, on 5% and 6% potassium chloride, only isolates from clades I and III exhibited exponential growth. In the presence of 2% sodium formate, only isolates from clade I grew exponentially (Fig. S1H, Table S2). Thus, isolates from clades I and III are less susceptible to osmotic stress than are clade II and IV isolates.

### The clade II isolate was highly sensitive to chemicals.

The phenotypic microarrays include five plates containing 120 different chemicals at 4 different concentrations, including antifungals (PM21 to PM25). All C. auris isolates failed to grow on 6 chemicals: BAPTA, an intracellular calcium chelator (15); methyl viologen dichloride, a transfer catalyst in redox reactions; protamine sulfate, a blood factor with antimicrobial properties (16, 17); thallium acetate, used as a selective growth agent in microbiology (18); thiourea, a known growth inhibitor of fungi (19); and trifluoperazine, an antipsychotic agent to treat schizophrenia (20) (Fig. S1I, J, and L). Exponential growth was detected on 114 chemicals in at least one isolate. The isolates from clades I and IV exhibited resistance to more chemicals than did clade III isolates (Fig. S1I to M, Table S2). Interestingly, the commonly used reference strain AR 0387 (B8441) was the least versatile of all tested clade I isolates, challenging the concept of studying individual isolates in depth rather than examining the broader versatility of multiple C. auris strains. Importantly, we detected almost no metabolic activity for the isolate of clade II (AR 0381) on most chemicals, since the strain grew only on five compounds (berberine, chlortetracycline hydrochloride, doxycycline, fumaric acid, and Niaproof) (Fig. S1K to M).

### Utilization of GlcNAc as nitrogen source was strain dependent.

**FIG 3:**
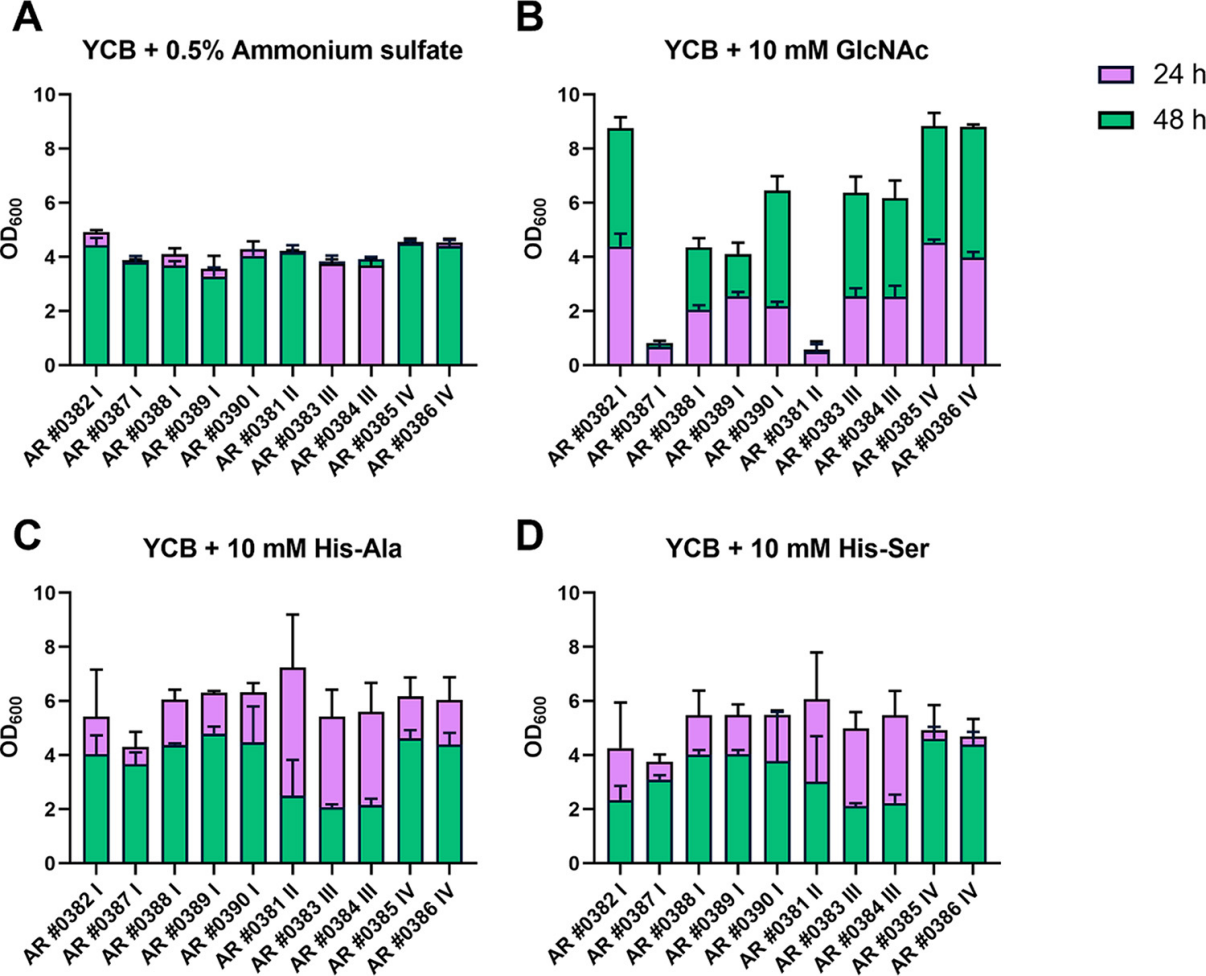
Growth curves of C. auris isolates on various nitrogen sources. SD overnight cultures were adjusted to an OD600 of 0.1 in YCB medium containing either 0.5% ammonium sulfate (A) or a 10 mM concentration of GlcNAc (B), His-Ala (C), or His-Ser (D) and incubated at 37°C with shaking. The OD600 was measured after 24 h and 48 h. The values shown are the calculated means and standard deviations of three biological replicates. Altogether, our growth experiments were consistent with the results of the phenotypic microarray screen.

Since the composition of the basic medium and the concentrations of the tested compounds in the phenotype microarrays were not disclosed, we next aimed to validate some of the results from the phenotypic screen. Therefore, we performed growth curve assays in defined media with selected nitrogen sources: GlcNAc, histidine-alanine (His-Ala), and histidine-serine (His-Ser). We also included ammonium sulfate as nitrogen source, which is not included in the phenotype microarray nitrogen sources plates but is used as a standard nitrogen source in the laboratory routine. The growth curve assays were performed in flasks to ensure an adequate distribution of oxygen. All strains showed similar growth in yeast carbon base (YCB) medium containing 0.5% ammonium sulfate as nitrogen source (Fig. 3A). Further, all C. auris isolates were able to utilize His-Ala and His-Ser as nitrogen source (Fig. 3C and D), which confirmed the results of the phenotype microarray screen (Fig. S1E and G). Growth on His-Ala reached a higher optical density at 600 (OD_600_), proving that this dipeptide is exceptionally well utilized by C. auris (Fig. 3C and D). Interestingly, while all isolates reached maximum growth on ammonium sulfate within 24 h (with average OD_600_s of ~4), growth on dipeptides was continuous and exceeded an OD_600_ of 4 for most isolates at 48 h (Fig. 3A, C, and D).

The clade II isolate AR 0381 was not able to utilize GlcNAc as nitrogen source in the growth curve assays (Fig. 3B), similar to what was noted in the phenotypic screen (Fig. S1C). However, both isolates from clade III grew well on GlcNAc as nitrogen source in the growth curve assay (Fig. 3B), which differed from the phenotypic microarray results where the isolates exhibited no exponential growth on GlcNAc (Fig. S1C). Interestingly, the growth behavior of isolates from clade I depended on the assay conditions. Isolate AR 0382 grew very well on GlcNAc (OD_600_, >8.75), whereas isolate AR 0387 showed only minor growth (OD_600_, ~0.8) in the growth curve assays (Fig. 3B). The other clade I isolates exhibited intermediate growth on GlcNAc as nitrogen source (Fig. 3B). The growth curves were performed in flasks, whereas the phenotypic screen was done in 96-well plates. Apart from the variation of the medium composition, this could also have contributed to the observed differences in growth.

### C. auris was impaired in utilization of monocarboxylic acids, whereas utilization of di- and tricarboxylic acids was clade and strain dependent.

**FIG 4:**
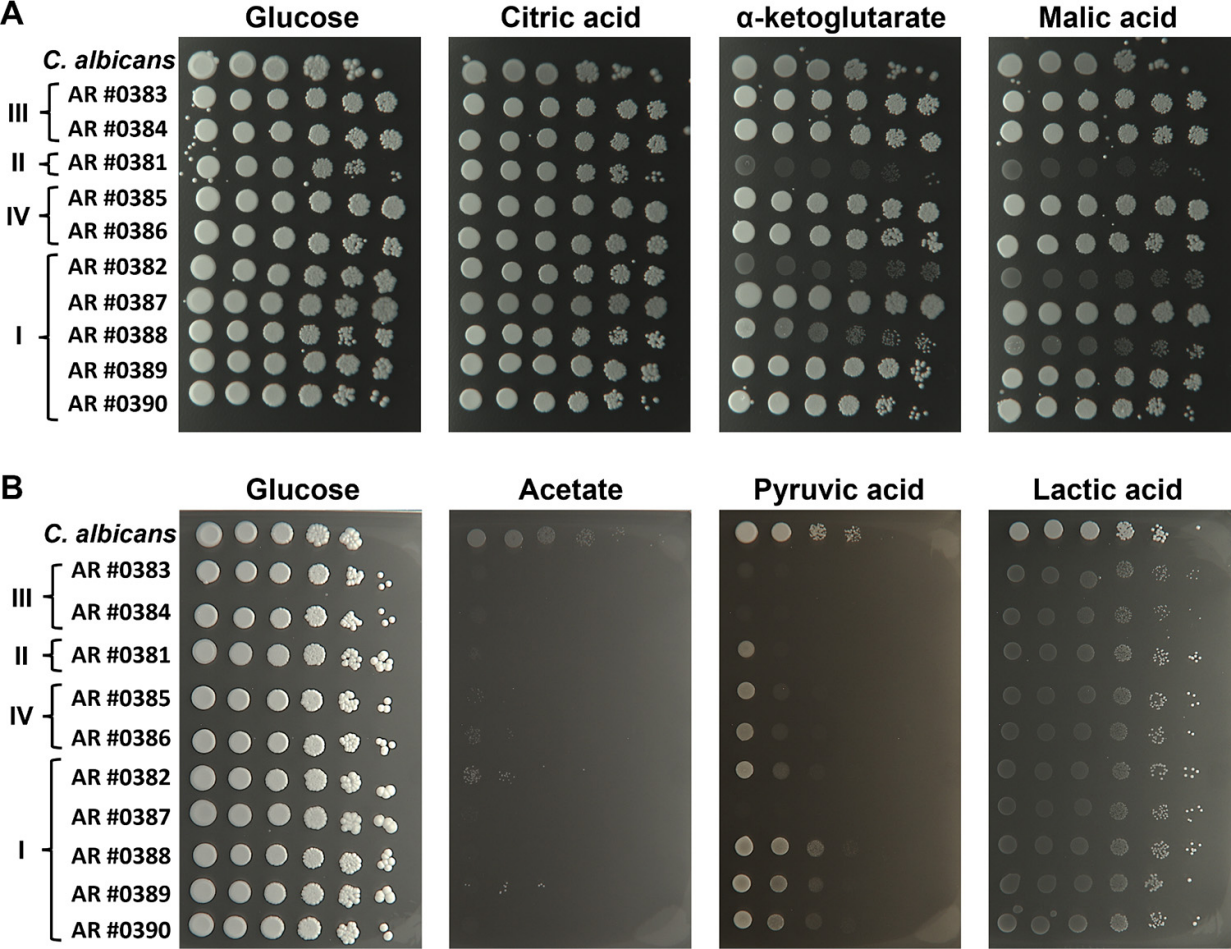
C. auris has an impaired ability to utilize monocarboxylic acids. SD overnight cultures of the strains were adjusted to an OD600 of 1.0. Serial 10-fold dilutions were spotted onto agar plates containing 1% glucose or 1% of the di- or tricarboxylic acid (A), and 1% glucose or 1% of the indicated monocarboxylic acid (B). Plates were incubated for 3 days at 37°C.

Carboxylic acids can be used as alternative carbon sources in many glucose-deficient host niches. While reviewing the phenotypic microarray results, we noted that many oxygen-dependent carbon sources, like pyruvate, lactic acid, or tricarboxylic acid (TCA) cycle intermediates, did not support exponential growth for all (α-ketoglutaric acid [α-KG], citric acid, succinic acid) or for some (malic acid, pyruvic acid) C. auris isolates (Fig. S1A). A similar effect has been reported for C. albicans (21), which is known to utilize carboxylic acids well. Therefore, we hypothesized that the phenotypic microarray plates did not provide sufficient aeration to support optimal growth on these carbon sources. The AUC values revealed that many isolates had some metabolic activity on TCA cycle intermediates, but these values fell below the exponential growth threshold (Table S2). Thus, we tested the growth capacity of all C. auris isolates on various alternative carbon sources by spot dilution assays and included C. albicans wild-type strain SC5314 for direct comparison. All strains exhibited growth on citric acid, whereas on α-ketoglutaric acid, succinic acid, and malic acid C. auris isolates from clade III (AR 0383, AR 0384), IV (AR 0385, AR 0386), and most isolates from clade I (AR 0387, AR 0389, AR 0390) showed robust and even more prominent growth than C. albicans (Fig. 4A). The clade II isolate (AR 0381) and two isolates from clade I (AR 0382, AR 0388) grew poorly on these carbon sources. Next, we tested the growth of the C. auris isolates on monocarboxylic acids, such as acetate, pyruvic acid, and lactic acid. As expected, C. albicans showed growth on all tested monocarboxylic acids (Fig. 4B). In contrast, all C. auris isolates failed to grow on acetate and displayed severe growth defects on lactic acid. On pyruvic acid as carbon source, only the isolates from clade III and one isolate from clade I (AR 0387) failed to grow (Fig. 4B). Thus, di- and tricarboxylic acids supported growth of C. auris, but their utilization was dependent on the clade and isolate, whereas all isolates were impaired in utilization of monocarboxylic acids.

### C. auris has no homolog of Jen1, a specialized monocarboxylate transporter.

**FIG 5:**
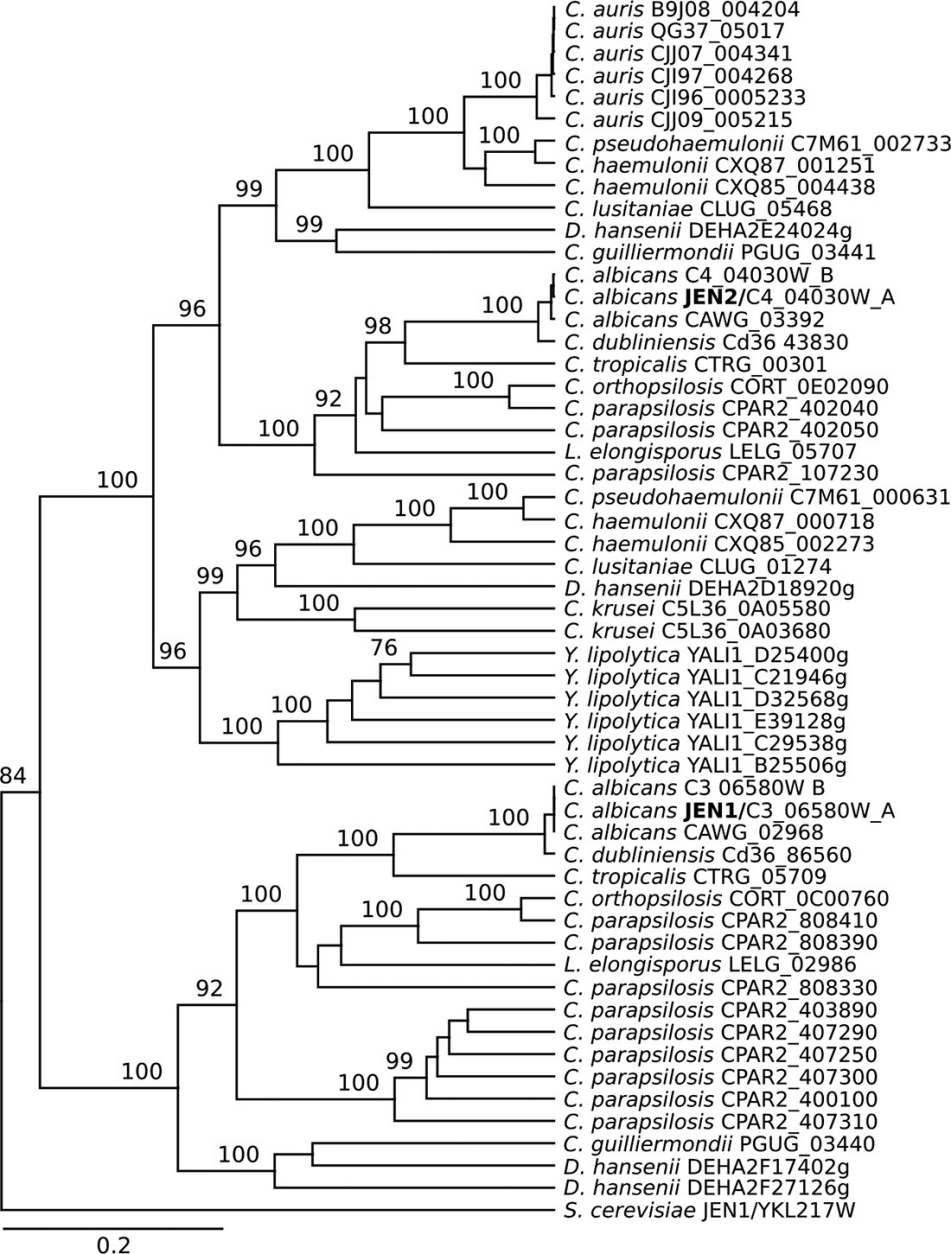
The phylogenetic tree of S. cerevisiae Jen1 homologs identified one homolog in C. auris. The phylogenetic tree of S. cerevisiae Jen1 homologs was generated by using the Galaxy platform to set up a BLAST database with public genome data followed by the identification of homologous sequences in C. auris and related species by applying the tblastn tool. The gene ID is given in the label (e.g., CAWG_03392).

In fungi, monocarboxylic acids can be transported via Jen1, a specialized and conserved transporter first reported in Saccharomyces cerevisiae (22–24). Most Candida spp. possess at least two S. cerevisiae Jen1 homologs, but only C. albicans Jen1 and Jen2 have been functionally characterized: Jen1 as a monocarboxylate transporter for lactate, propionate, and pyruvate and Jen2 as a dicarboxylate transporter for succinate and malate (21, 25, 26). We analyzed the genomes of all publicly available genomes for several C. auris isolates of different clades and detected only one homolog of S. cerevisiae Jen1 in C. auris (B9J08_004204), which clustered closer to C. albicans Jen2 than C. albicans Jen1 in a phylogenetic analysis (Fig. 5). The lack of a specialized monocarboxylate transporter could explain why C. auris can utilize dicarboxylic acids but cannot grow well on monocarboxylic acids.

### Growth on alternative carbon sources elicited both common and unique transcriptional responses in C. auris and C. albicans.

**FIG 6:**
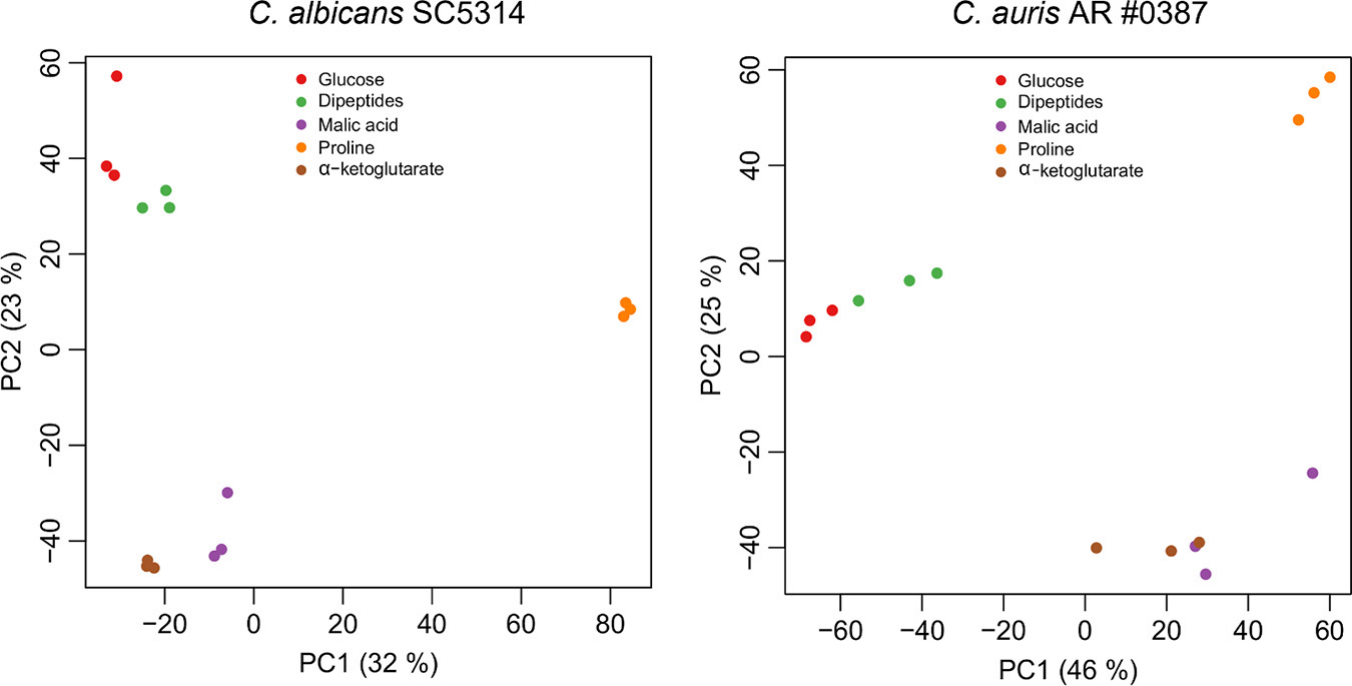
Principal-component analysis of C. albicans SC5314 and C. auris AR 0387. Transcriptional profiling was performed with C. albicans and C. auris cells grown on different carbon sources (glucose, malic acid, α-ketoglutarate, proline) or on a mixture of dipeptides (3.4 mM Ala-Gln, 3.3 mM Ala-Ser, 3.3 mM Arg-Asp) as a nitrogen source. Each color represents a different medium condition of three biological replicates of C. albicans strain SC5314 and C. auris strain AR 0387.

**FIG 7:**
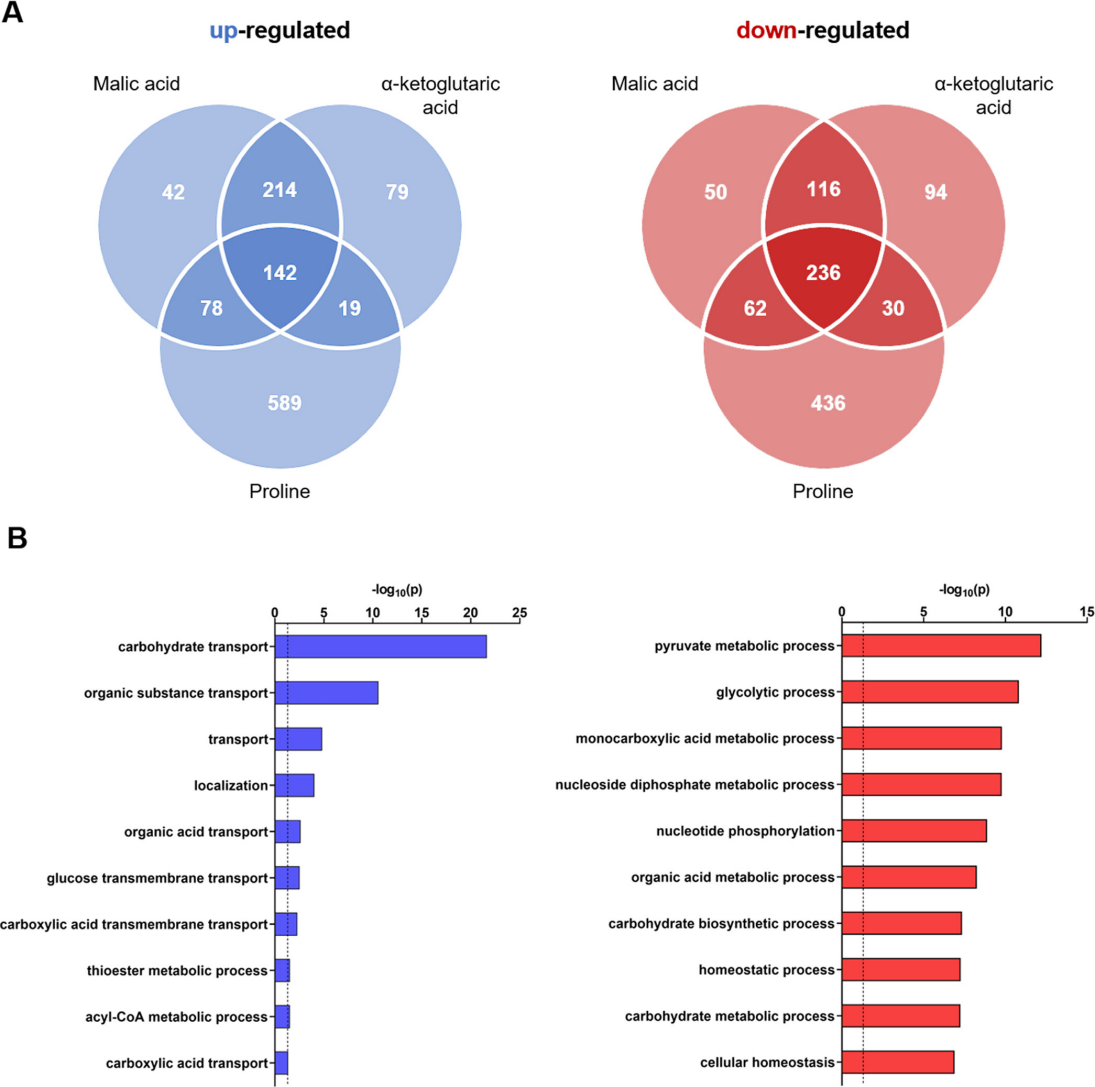
Transcriptional response of C. albicans strain SC5314 to malic acid, α-ketoglutaric acid, and proline as a C source. (A) Venn diagrams representing all significantly differentially regulated genes with a log2 fold change of >1 (blue) and <(−1) (Zamith-Miranda [45]) and a *P* value of <0.05. (B) Gene ontology analyses of the upregulated (blue, *n* = 142) and downregulated (red, *n* = 236) core sets of genes were performed by using the GO term finder and with “process” as a query, followed by using the Revigo tool (http://revigo.irb.hr/). Only the 10 most significant enriched GO terms, defined by a −log10 value of 0.05, are shown and are indicated by a dashed line at 1.3 on the x axis.

**FIG 8:**
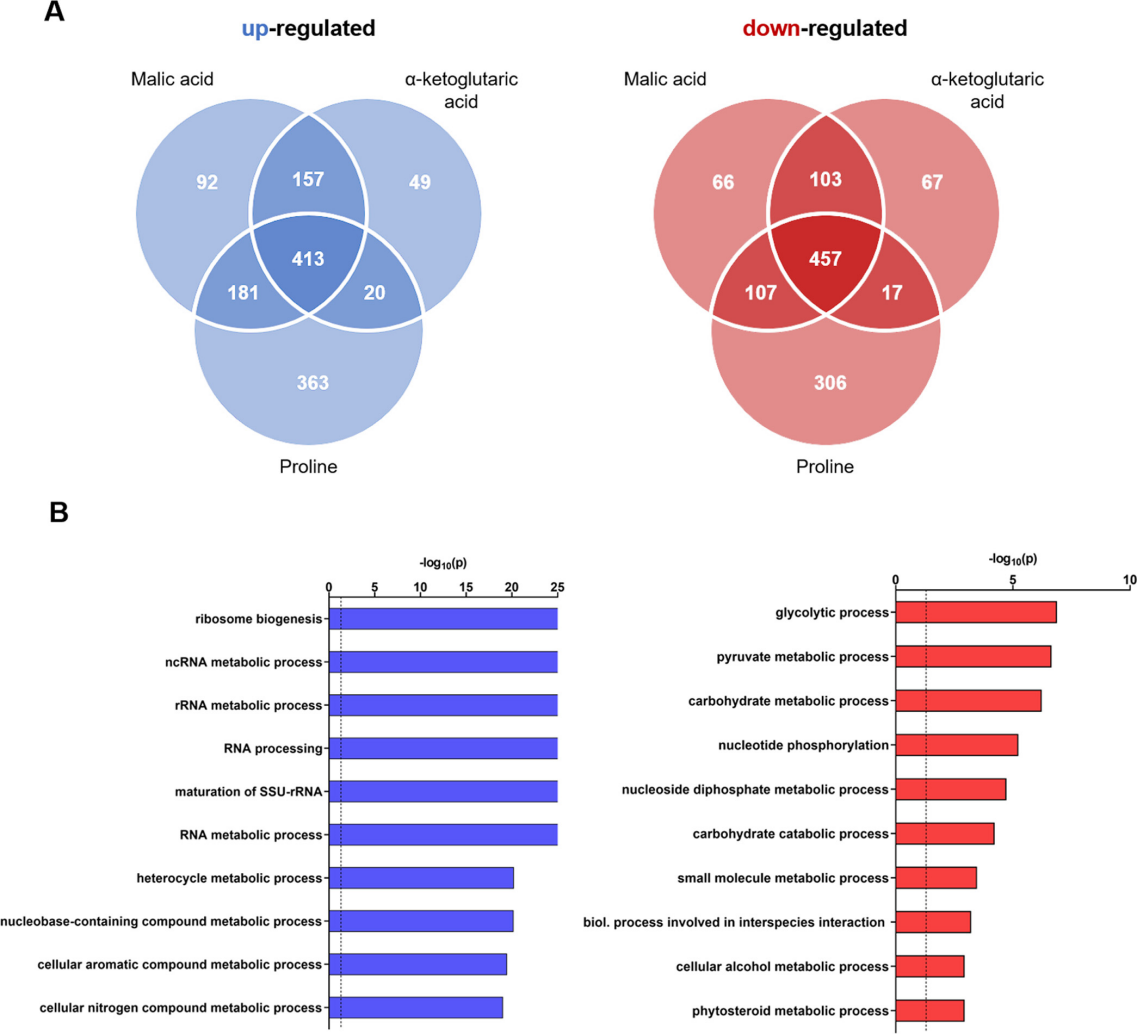
Transcriptional response of C. auris strain AR 0387 to malic acid, α-ketoglutaric acid, and proline as C sources. (A) Venn diagrams representing all significantly differentially regulated genes with a log2 fold change of >1 (blue) and <(−1) (Zamith-Miranda [45]) and a *P* value of <0.05 (blue). (B) Gene ontology analyses of the upregulated and downregulated core sets of genes that have an ortholog in C. albicans were performed by using the GO term finder and “process” as a query, followed by using the Revigo tool (http://revigo.irb.hr/). Only the 10 most significant enriched GO terms, defined by a −log10 of 0.05, are shown (indicated by a dashed line at 1.3 on the x axis).

Since C. auris and C. albicans show substantial differences in utilization of alternative carbon sources and C. auris grows well on dipeptides as the sole nitrogen source, we next asked what transcriptional changes accompany growth on alternative carbon sources and dipeptides in both species. To answer this question, we selected three carbon sources that are tightly connected to the TCA cycle and support equally well the growth of C. auris and C. albicans: malic acid, α-KG, and proline. For the dipeptide medium, we used a mixture of Ala-Gln, Ala-Ser and Arg-Asp as a nitrogen source. The transcriptional changes were compared to cells grown in glucose as carbon source and ammonium sulfate as nitrogen source (control condition here referred to as “glucose”). We observed clear medium-specific effects in both species with three distinct clusters: cells grown in malic acid and α-KG; cells grown in glucose and dipeptides; and cells grown in proline (Fig. 6). These effects were reflected in the extensive overlap of gene expression changes between cells grown in malic acid and α-KG (Fig. 7A and 8A) and the relatively small transcriptional changes in cells grown on dipeptides compared to the control condition.

Interestingly, C. auris elicited a more robust response to the tested alternative carbon sources compared to C. albicans with 1,275 upregulated and 1,123 downregulated genes under the three conditions (Fig. 8A). In comparison, the observed changes in C. albicans yielded 1,163 upregulated genes and 1,024 downregulated genes in response to the tested carbon sources (Fig. 7A).

The responses of both species to the different conditions had certain overlaps, and in both species there was a set of genes with changed expression under all three growth conditions compared to the control. For instance, the general ontology (GO) term analysis identified for both species genes related to ribosome biogenesis and RNA processing among the induced genes in each alternative carbon source (Fig. 7B and 8B). Further, there was a substantial number of induced genes that related to carboxylic acid and organic substance transport and metabolism. C. albicans JEN2 and its homolog in C. auris B9J08_004204 were consistently highly upregulated in all carbon sources. From the downregulated genes, the category of metabolic process or biogenesis was found in all cases (Fig. 7B and 8B). Thus, both species responded by inducing RNA processing and metabolism, transport of organic compounds, and downregulation of various metabolic processes related to the switch from preferred to nonpreferred nutrient.

The GO term analysis also identified species-specific and nutrient-specific categories. On malic acid, for example, C. auris induced a large category of DNA replication and synthesis genes that was absent under the other conditions and in C. albicans grown on this nutrient (Tables S3 and S4). From the downregulated genes on malic acid, there was a substantial number of genes responding to oxidative stress, a category absent in C. albicans, and in the other nutrients. C. albicans growth on malic acid resulted in downregulation on trehalose metabolism and response to or detoxification of toxic substances. The transcriptional changes in C. auris and C. albicans that were specific to α-KG included the induction of organic compound transport, mitochondrial membrane organization, and transport. Growth on proline, which elicited the largest transcriptional changes in both species, induced genes involved in regulation of α-amino acid metabolic processes in C. auris and genes of fatty acid metabolism and alternative carbon source metabolism in C. albicans. The downregulated genes were related to the carbohydrate derivative response to stress and regulation of biological process in C. auris and carbohydrate and steroid biosynthesis in C. albicans. Thus, we were able to identify both common and unique responses to nonpreferred carbon sources in both species.

### Predicted C. auris transporters with no C. albicans ortholog are on top of the list of highly regulated genes.

The response to dipeptides resulted in the upregulation of only 127 genes and downregulation of 57 genes in C. auris, whereas in C. albicans 277 genes were upregulated and 259 genes were downregulated. Many genes were involved in regulating transport and metabolism of organic substances (Tables S3 and S4). Notable, while the transcriptional changes in C. auris were not as prominent, 30 upregulated and 24 downregulated genes had no C. albicans ortholog, a substantial number based on the overall changes in transcription. Four of those had as a best hit OPT1 (B9J08_004062, B9J08_004066) and OPT3 (B9J08_004560, B9J08_004538) in C. albicans, which, along with the *OPT1* ortholog B9J08_004602, were the only induced genes from this oligopeptide transporter family (Table S5). C. albicans grown on dipeptides as nitrogen source resulted in upregulation of *OPT1-4* but downregulation of *OPT7* and *OPT8*. The di- and tripeptide transporter-encoding gene *PTR22* in C. albicans and the orthologs or best hit in C. auris, B9J08_004188, B9J08_003830, and B9J08_005124, were not differentially expressed. Thus, while *OPT1* and *OPT3* and their orthologs or best hits were highly induced in both species in response to dipeptides as nitrogen source, the dipeptide- and tripeptide-encoding genes remained transcriptionally unchanged.

Our analysis showed that many differentially regulated genes in C. auris grown on alternative carbon sources also lacked a C. albicans ortholog or best hit match (Table S4). Specifically, 98, 73, and 136 C. auris-specific genes were upregulated and 132, 148, and 148 genes were downregulated in malic acid, α-KG, and proline, respectively. Importantly, many of these genes appeared to be among the most highly regulated ones and had predicted roles in transport or metabolism of nutrients. We found particularly interesting the presence of several C. auris genes that had the C. albicans nicotinic acid transporter gene TNA1 as ortholog or best hit. Indeed, we found five C. auris genes similar to TNA1 in C. albicans: one ortholog and four best hits. Additionally, we identified six other genes that had as ortholog or best hit TNA1 in S. cerevisiae, while in C. albicans the same genes had orf19.3232 and VHT1 as orthologs or best hits. Of the 11 TNA1-like genes in C. auris, B9J08_005570 and B9J08_005571 were nearly identical, and along with B9J08_002974, B9J08_004243, and B9J08_004893 were highly induced in all tested carbon sources compared to glucose (Tables S3 and S4). The VHT1 ortholog (B9J08_002974), and best hit (B9J08_004448) genes were significantly downregulated on carbon sources, whereas the B9J08_005572 gene remained unchanged (Tables S3, S4, and S5). All 11 genes were either downregulated or not changed in dipeptides. Thus, C. auris TNA1 transporters have expanded in number and appear to carry functions related to metabolism of alternative carbon sources.

## DISCUSSION

In the past decade, the fungal pathogen C. auris has caused multiple outbreaks around the world. As this pathogen is not only difficult to treat but also difficult to identify, it is imperative to make steps toward achieving rapid identification and containment of this species. Thus, we need to understand in depth its ecology, diversity, growth characteristics, and interactions with the human host. To date, while many studies have focused on C. auris genomics and antifungal resistance, knowledge of the phenotypic characteristics of this fungus remains limited.

In this study, we performed a comprehensive phenotypic profiling of C. auris isolates from the four main clades. With this effort, we significantly expanded the list of nutrients and chemicals that support C. auris growth and helped to identify multiple clade- and species-specific phenotypic variations. We and others have shown that clade II isolates are unique in many aspects. For instance, the isolate from clade II, AR 0381, showed the lowest capability to use carbon sources, did not tolerate high osmolyte concentrations, and most importantly, grew on less than 10% of the chemicals that were tolerated by the other clades. On the other hand, this was the only clade capable of exponential growth on sorbic acid as a carbon source or Arg-Phe and Leu-Leu dipeptide combinations and utilized preferentially tetramethylene sulfone as a sulfur source and alanine-containing dipeptides as a nitrogen source. Isolates from clade II have been isolated most frequently from the ear canal and exclusively from individuals in Japan and Korea. Asian population tends to have a dry type of ear cerumen compared to non-Asian populations (27). The nutrient composition of the ear cerumen includes various nitrogen sources (proline, phenylalanine, tryptophan, tyrosine) and carbon sources (squalene, long-chain fatty acids, diterpenoids) (28–30). Thus, we assume that the clade II isolates must have adapted to this specific host niche, which has dictated the development of their unique phenotypic properties and noninvasive clinical presentation. Although the number of isolated strains from clade II is limited, further studies would validate if the observed growth characteristics of strain AR 0381 are clade specific.

We were also able to confirm previously reported phenotypes, such as osmotolerance and clade- or strain-specific resistance to antifungals and other chemicals. Indeed, previous studies showed that this fungus is able to tolerate higher salt concentrations (10% [wt/vol] NaCl) compared to other *Candida* species (13, 14, 31). In our screen, we noted uniform tolerance to high concentrations of sodium nitrate, sodium phosphate, and ammonium sulfate, but with other osmolytes the clades separated into two clear groups, since clades I and III showed much higher resistance to osmotic stressors than clades II and IV. Interestingly, only clade I retained active growth when 6% NaCl was combined with other osmolytes, such as trehalose, glycerol, and triethylamine. This implies that clade I isolates have the highest capacity to withstand harsh conditions, such as the reported ability to persist on medical equipment, hospital bed space areas, and human skin (2, 13, 32, 33). In addition, representatives of this clade have been isolated from marine wetlands (34, 35), which may have driven the development of osmotolerant characteristics.

Application of combinatorial stress or therapy is one approach to eradicate C. auris persistent infections and colonization of hospital units. It is now well established that this species is relatively resistant to certain combinatorial stresses (alkaline pH and 47°C), but sensitive to common hospital linen laundering protocols (alkaline pH and >80°C) (36). Thus, it is critical to identify the combination(s) of conditions that can inhibit C. auris growth most effectively. In this study, we tested growth on 120 different chemicals, including antifungal agents. Of these, only 6 chemicals inhibited the growth of all tested isolates: BAPTA, methyl viologen dichloride, protamine sulfate, thallium acetate, thiourea, and Trifluoperazine. However, BAPTA, methyl viologen dichloride, thallium acetate, and thiourea are either toxic or carcinogenic for humans. In contrast, trifluoperazine is an antipsychotic used to treat schizophrenia (20) and protamine sulfate is used to neutralize the effect of heparin during delivery and heart surgery (17). Further, protamine sulfate can suppress the expression of virulence proteins of Pseudomonas aeruginosa (16). Thus, trifluoperazine or protamine sulfate could be potential candidates for targeting C. auris growth either by themselves or in combination with other antifungal agents. To date, various combinations of two antifungal agents or an antifungal and an antibacterial or antiparasitic agent, chemical, or natural product have been tested (37, 38). For instance, the combination of caspofungin and the nucleoside antibiotic nikkomycin Z result in a weak to strong synergistic antifungal effect (38). In C. albicans the di- and tripeptide transporter Ptr22 facilitates uptake of nikkomycin Z into the cell (39). Interestingly, clade III isolates were less sensitive to nikkomycin Z than others, which goes well with our observation that isolates from clades III and IV have a more limited repertoire of growth-supporting dipeptides compared to the other clades (40). C. auris has an expanded number of peptide transporters that not only support the utilization of more Glu- and Gly-containing dipeptides compared to C. albicans (39) but also show elevated expression levels upon exposure to conventional antifungals (4, 41, 42). Others, like the ortholog or best hit of C. albicans di- and tripeptide transporter Ptr22, were not regulated on a transcriptional level in our data sets, consistent with previous observations (39). The substrate specificities of these unique transporters are yet to be revealed.

In addition to dipeptides, GlcNAc as a nitrogen source supported the exponential growth of almost all tested isolates except the clade II isolate. While clade-specific differences in GlcNAc assimilation as a carbon source have been previously reported (43), the differences in the utilization of this nutrient as a nitrogen source have not yet been investigated. The clade II isolate did not grow on GlcNAc as either a carbon or nitrogen source. Interestingly, both isolates from clade III showed exponential growth only when GlcNAc was provided as carbon source, but not when it was used as nitrogen source in the phenotypic screen. However, in our growth curve analysis, both clade III isolates grew well on GlcNAc as a nitrogen source, suggesting that the ability of C. auris to utilize this nitrogen source may depend on the concentration and/or the oxygen levels. The growth curves were performed in flasks, whereas the phenotypic screen was performed in 96-well plates. It is also feasible to speculate that such differences may arise from the differential rate or efficiency of uptake, incapacity to withstand the accumulation of toxic metabolic byproducts, or from clade-specific genome modifications (44).

C. auris isolates either failed (acetate) or grew very poorly (lactic acid) on monocarboxylic acids as carbon source, whereas C. albicans showed robust growth on monocarboxylic acids. The transport of monocarboxylic acids in fungi is mediated by the conserved transporter Jen1, which was first described in S. cerevisiae (22–24). C. albicans genome encodes two homologs of S. cerevisiae Jen1: a monocarboxylate and a dicarboxylate plasma membrane transporter named Jen1 and Jen2, respectively (21, 25). According to Alves et al., the C. auris genome encodes two Jen homologs of the S. cerevisiae Jen1 transporter (26). However, our careful analysis of multiple C. auris isolates showed that the genome of this species encodes only one homolog per genome which is phylogenetically related to the C. albicans dicarboxylic acid transporter Jen2. Thus, the lack of the monocarboxylic acid transporter Jen1 in C. auris could explain the observed growth defect on monocarboxylic acid as a carbon source.

Another metabolic difference between C. auris and C. albicans is the utilization of di- and tricarboxylic acids. Most C. auris isolates from clade I and both isolates from clade III and IV exhibited more robust growth on di- and tricarboxylic acids than the C. albicans wild-type strain SC5314. Such robust growth might be linked to the previously observed high abundance of enzymes from the TCA cycle (45) or due to a more efficient transport of these nutrients. Furthermore, C. auris also elicited a more robust, though similar, transcriptional response compared to C. albicans following grown on malic acid, α-ketoglutaric acid, and proline. One of the most highly induced genes in both species was *JEN2*, and its ortholog in C. auris, B9J08_004204. C. auris growth on alternative carbon sources is related to mitochondrial function and resulted in the induction of a significant number of species-specific genes, for example, genes without an exact C. albicans ortholog. Of those, the best hit was a gene encoding the nicotinic acid transporter Tna1 in C. albicans. Although the specificity of these potential transporters is currently unknown, it is possible that some of them mediate the utilization of alternative carbon sources and contribute to the robust utilization of dicarboxylic acids. Increased ATP production and reduced oxidative stress during respiratory growth have been shown to be important for fluconazole resistance in C. albicans (46). Thus, it is possible that the enhanced respiration, or the additional transporters in C. auris, are linked to the antifungal resistance in this species. Further work can shed light on the important processes that C. auris gene expansions serve and the regulatory mechanisms involved in their activation.

## MATERIALS AND METHODS

### Strain cultivation.

All strains used in this study are listed in Table S1. All strains were stored as frozen stocks containing 20% glycerol at −80°C and subcultured on YPD agar plates (1% yeast extract, 2% peptone, 2% glucose, 2% agar) at 30°C for 2 days. Strains were routinely grown in YPD liquid medium at 30°C overnight with shaking at 180 rpm.

### High-throughput phenotypic screen.

C. auris strains were pregrown on YPD agar plates incubated at 30°C for 24 h. The high-throughput phenotypic screen was performed using Biolog Phenotype MicroArrays for microbial cells (PMs) according to the manufacturer’s protocol for yeasts (Biolog, Inc., Hayward, CA, USA), and as reported previously (39). The following plates were used: PM1, PM2 (carbon sources), PMs 3, 6, 7, and 8 (nitrogen sources), PM4 (phosphorus and sulfur sources), PM9 (osmolytes), and PMs 21 to 25 (chemical sensitivity). The plates were incubated at 37°C for 24 to 48 h in an OmniLog plate reader (Biolog, Inc., Hayward, CA, USA), and metabolic activity was recorded automatically every 15 min at an OD of 750 nm.

### Statistical analysis.

The respective negative control, if present, was subtracted from the growth signals in each Phenotype MicroArray. Negative values were replaced by zero, if present. Next, each array’s growth signals were categorized into two groups: exponential growth (active growth) and no exponential growth (nonactive growth), as proposed by Vehkala et al. (47). In brief, a data-fitted logistic curve represented exponential growth, while a line without showing exponential growth phase characteristics was interpreted as nonactive growth in the investigated timeframe. The method was repeated for each replicate separately. Hence, a given substrate’s growth signal was detected as exponential if at least two replicates were detected active. Next, the growth signals were normalized as proposed by Vehkala et al. (47). In brief, the middle growth curve among triplicates was taken as a reference, and the other two nonreferenced growth curves were multiplied by the calculated normalization factor to reduce the systematic errors in the experiments. As a metric of growth strength, the AUC of normalized signals was calculated over time points per substrate per phenotypic microarray assay. Grouping and normalizing the growth signals were implemented in R version 4.1.2 with the pipeline proposed by Vehkala et al. (47), building upon the opm package version 1.3.77 (48).

### Growth assays.

Growth curves in liquid media were performed in YCB medium (11.7 g/liter yeast carbon base; pH 5.0) containing either 0.5% ammonium sulfate or 10 mM *N*-acetylglucosamine, His-Ala, or His-Ser. Overnight cultures in synthetic dextrose (SD) medium (0.17% (wt/vol) yeast nitrogen base without ammonium sulfate and amino acids, 0.5% (wt/vol) ammonium sulfate, 2% (wt/vol) glucose) of the respective C. auris strains were centrifuged (4,000 × *g*, 5 min), and washed once with distilled H_2_O (dH_2_O). Strains were adjusted to an OD_600_ of 0.1 in 13 mL YCB medium containing the indicated nitrogen sources and incubated for 48 h at 37°C with shaking at 180 rpm. The OD_600_ was measured after 24 h and 48 h.

### Spot dilution assay.

SD overnight cultures of the respective strains were centrifuged (4,000 × g, 5 min) and washed once with dH2O. The cell density was adjusted to an OD600 of 1.0, and 5 μL of each 10-fold serial dilution was spotted on YNB agar plates (0.17% [wt/vol] yeast nitrogen base without ammonium sulfate and amino acids, 0.5% [wt/vol] ammonium sulfate, 2% agar) containing 1% α-ketoglutaric acid, citric acid, glucose, lactic acid malic acid, pyruvic acid, sodium acetate, or succinic acid. The plates were incubated for 3 days at 37°C.

### Evaluation of genetic differences.

To investigate differences observed in the phenotypic assay, we explored the frequency and/or sequence of candidate genes potentially involved in the clade differences. Candidate genes and proteins that originated from known pathways in Saccharomyces cerevisiae or Candida albicans served as query for BLAST analyses against a set of publicly available C. auris genomes representing the different clades. Therefore, we utilized the Galaxy platform (https://usegalaxy.org/, version 21.09.1.dev0) as follows: a BLAST database (Galaxy Tool ID; https://toolshed.g2.bx.psu.edu/repository?repository_id=1d92ebdf7e8d466c) was set up and the blastn or tblastn tool was used to identify homologous sequences in C. auris. Hits were evaluated manually, and phylogenetic trees were constructed.

### Transcriptional profiling.

**(i) RNA isolation.** YPD overnight cultures of the C. albicans SC5314 and C. auris AR 0387 strains were centrifuged (4,000 × g, 5 min) and washed twice with phosphate-buffered saline (PBS). The cell density was adjusted to an OD600 of 0.2 in 30 mL SD medium (0.17% [wt/vol] yeast nitrogen base without ammonium sulfate and amino acids, 0.5% [wt/vol] ammonium sulfate, 2% [wt/vol] glucose), and cultures were incubated for 5 to 6 h at 37°C with shaking at 180 rpm. After the logarithmic growth phase was reached, the cells were centrifuged (4,000 × g, 5 min) and washed twice with PBS. The cell density was adjusted to an OD600 of 0.2 in 30 mL in YNB medium (0.17% [wt/vol] yeast nitrogen base without ammonium sulfate and amino acids, 0.5% [wt/vol] ammonium sulfate, 1% glucose [wt/vol]; pH 5) as control medium. For testing on different carbon sources, glucose was substituted with either 1% malic acid (Sigma-Aldrich, St. Louis, MO, USA), 1% α-ketoglutarate (Sigma-Aldrich), or 1% proline (Carl Roth, Germany). For testing on different nitrogen sources, ammonium sulfate was substituted with 10 mM dipeptides (3.4 mM Ala-Gln, 3.3 mM Ala-Ser, 3.3 mM Arg-Asp; Bachem, Switzerland). The cells were incubated in the respective media for 4 h at 37°C with shaking at 180 rpm. After incubation, cells were harvested by centrifugation (4,000 × g, 5 min), and cell pellets were frozen in liquid nitrogen. RNA isolation was performed as described previously (49). RNA quality and quantity were determined using the RNA Nano 6000 assay kit of the Bioanalyzer 2100 system (Agilent Technologies, CA, USA) and the NanoPhotometer spectrophotometer (IMPLEN, CA, USA), respectively.

**(ii) RNA sequencing.** RNA sequencing was performed by Novogene Europe (Cambridge, United Kingdom) with 150-bp paired ends and a minimum of 10 million reads per sample using the Illumina NovaSeq 6000 sequencing system. To prepare an RNA library, the mRNA was enriched by a poly(A) capture followed by reverse transcription of cDNA. Illumina PE150 technology was used for 150-bp paired-end sequencing.

**(iii) Bioinformatic analysis.** FastQC (version 0.11.9) was used to confirm quality of raw data. Reference genomes (*C_albicans*_SC5314_version_A22-s07-m01-r44_chromosomes.fasta and C_auris_B8441_version_s01-m01-r21_chromosomes.fasta) and genome annotations (*C_albicans*_SC5314_version_A22-s07-m01-r44_features.gtf and C_auris_B8441_version_s01-m01-r21_features.gtf) were downloaded from the *Candida* Genome Database. Paired-end reads were mapped to the respective reference genomes using Bowtie 2 (version 2.3.5.1). Mapped reads were assigned to genomic features using the function featureCounts of the R package Rsubread (version 2.2.6). Contrasts between sample groups were calculated using R package DESeq2 (version 1.28.1), and *P* values were adjusted according to the Benjamini-Hochberg method. C. albicans orthologs of C. auris were used for GO analysis of C. auris results. Ortholog information was downloaded from the Candida Genome Database (information was retreived from file C_auris_B8441_C_albicans_SC5314_orthologs.txt).

**(iv) Gene ontology and Venn analysis.** The expression profiles of the analyzed contrasts were filtered by setting a threshold of a log2 fold change of >1 or <(−1) and a *P* value of <0.05. Venn analyses were performed by using the Bioinformatics & Evolutionary Genomics online tool (http://bioinformatics.psb.ugent.be/webtools/Venn/). Gene Ontology analyses were performed by using the Candida Genome Database (CGD) GO term finder and “process” as a query, followed by using the Revigo online tool (http://revigo.irb.hr/). Graphical visualizations of GO term analyses were done by using GraphPad Prism version 9.4.

### Data availability.

The transcriptomic data discussed in this publication have been deposited in NCBI's Gene Expression Omnibus and are accessible through GEO series accession number GSE223412.
