# Supplementary material for: High-Throughput Profiling of Candida auris Isolates Reveals Clade-Specific Metabolic Differences
Source: Microbiol Spectr. 2023 Apr 25;11(3):e00498-23. doi: 10.1128/spectrum.00498-23 (PMC10269459; doi:10.1128/spectrum.00498-23)
Supplement: Supplemental file 1 — Supplemental material. Download spectrum.00498-23-s0001.pdf, PDF file, 9.3 MB [file spectrum.00498-23-s0001.pdf]

Supplemental material

Figure S1: Biolog Phenotype MicroArray results.

Table S1: Strains used in this study.

Table S2: Biolog Phenotypic MicroArrays analysis.

Table S3: *C. albicans* transcriptomic data.

Table S4: *C. auris* transcriptomic data.

Table S5: Transcriptionally induced gene families in *C. auris*

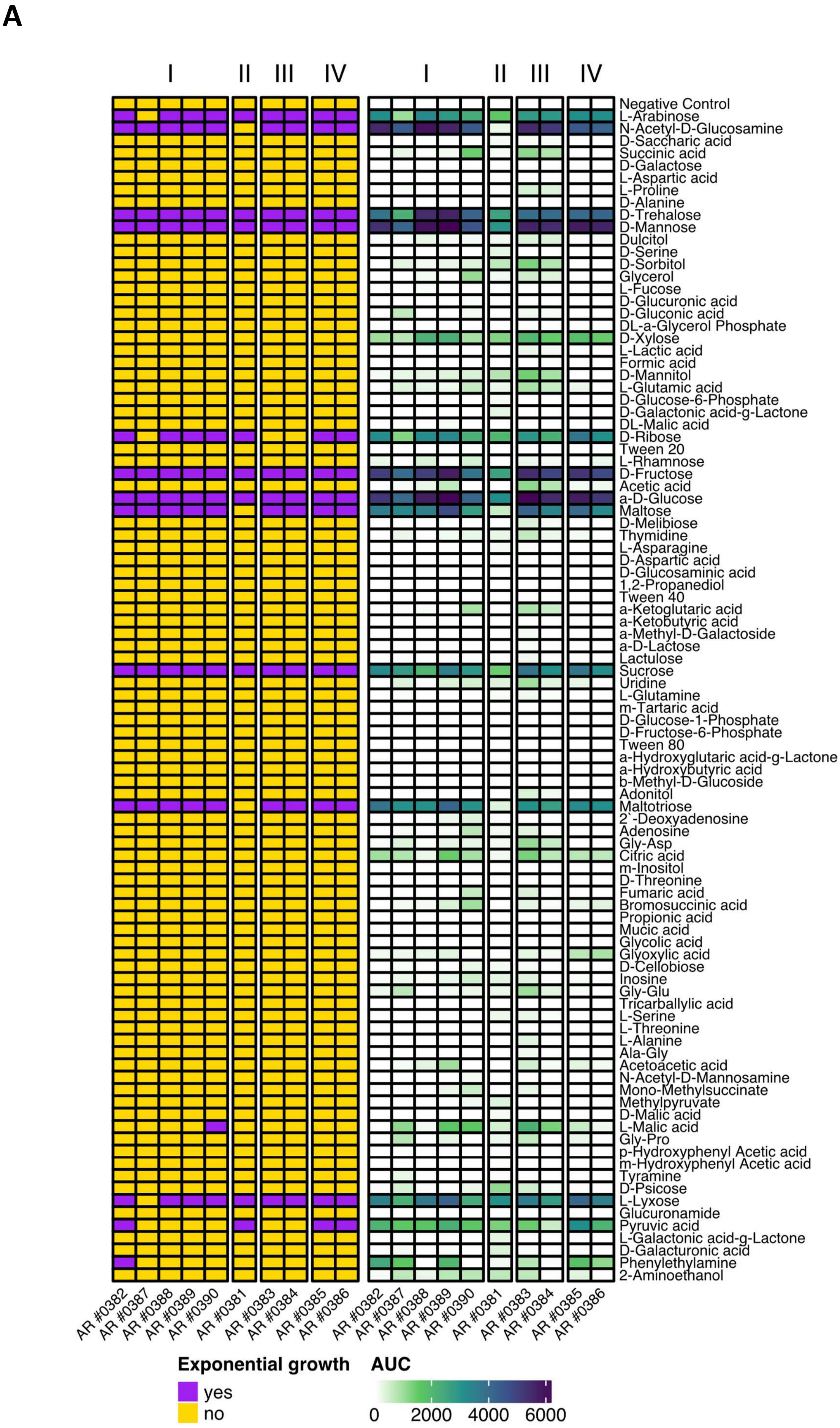
A
I
II
III
IV
I
II
III
IV
Negative Control
L-Arabinose
N-Acetyl-D-Glucosamine
D-Saccharic acid
Succinic acid
D-Galactose
L-Aspartic acid
L-Proline
D-Alanine
D-Trehalose
D-Mannose
Dulcitol
D-Serine
D-Sorbitol
Glycerol
L-Fucose
D-Glucuronic acid
D-Gluconic acid
DL-a-Glycerol Phosphate
D-Xylose
L-Lactic acid
Formic acid
D-Mannitol
L-Glutamic acid
D-Glucose-6-Phosphate
D-Galactonic acid-g-Lactone
DL-Malic acid
D-Ribose
Tween 20
L-Rhamnose
D-Fructose
Acetic acid
a-D-Glucose
Maltose
D-Melibiose
Thymidine
L-Asparagine
D-Aspartic acid
D-Glucosaminic acid
1,2-Propanediol
Tween 40
a-Ketoglutaric acid
a-Ketobutyric acid
a-Methyl-D-Galactoside
a-D-Lactose
Lactulose
Sucrose
Uridine
L-Glutamine
m-Tartaric acid
D-Glucose-1-Phosphate
D-Fructose-6-Phosphate
Tween 80
a-Hydroxyglutaric acid-g-Lactone
a-Hydroxybutyric acid
b-Methyl-D-Glucoside
Adonitol
Maltotriose
2`-Deoxyadenosine
Adenosine
Gly-Asp
Citric acid
m-Inositol
D-Threonine
Fumaric acid
Bromosuccinic acid
Propionic acid
Mucic acid
Glycolic acid
Glyoxylic acid
D-Cellobiose
Inosine
Gly-Glu
Tricarballylic acid
L-Serine
L-Threonine
L-Alanine
Ala-Gly
Acetoacetic acid
N-Acetyl-D-Mannosamine
Mono-Methylsuccinate
Methylpyruvate
D-Malic acid
L-Malic acid
Gly-Pro
p-Hydroxyphenyl Acetic acid
m-Hydroxyphenyl Acetic acid
Tyramine
D-Psicose
L-Lyxose
Glucuronamide
Pyruvic acid
L-Galactonic acid-g-Lactone
D-Galacturonic acid
Phenylethylamine
2-Aminoethanol
AR #0382
AR #0387
AR #0388
AR #0389
AR #0390
AR #0381
AR #0383
AR #0384
AR #0385
AR #0386
AR #0382
AR #0387
AR #0388
AR #0389
AR #0390
AR #0381
AR #0383
AR #0384
AR #0385
AR #0386
Exponential growth
yes
no
AUC
0
2000
4000
6000

B

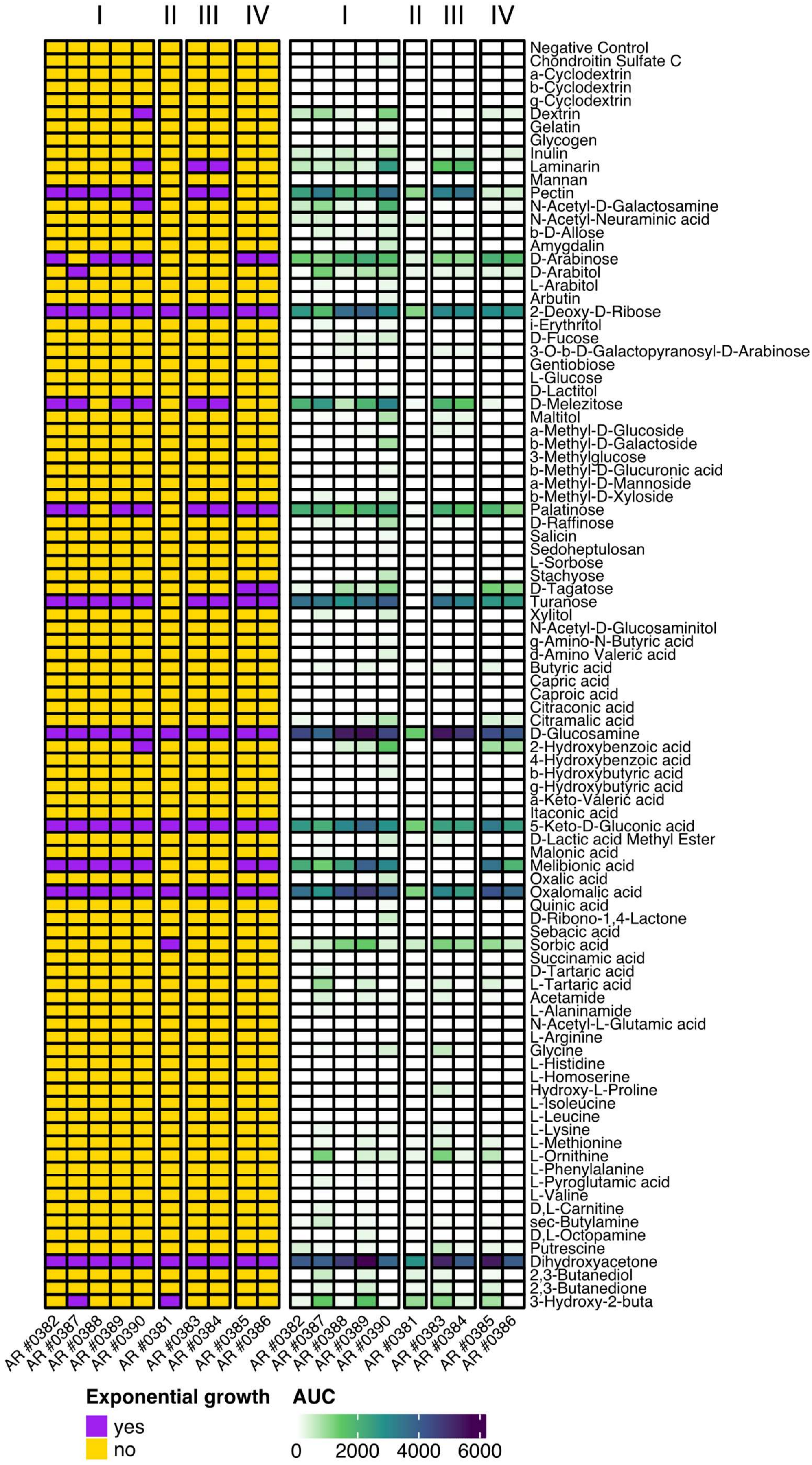
I
II
III
IV
I
II
III
IV
Negative Control
Chondroitin Sulfate C
a-Cyclodextrin
b-Cyclodextrin
g-Cyclodextrin
Dextrin
Gelatin
Glycogen
Inulin
Laminarin
Mannan
Pectin
N-Acetyl-D-Galactosamine
N-Acetyl-Neuraminic acid
b-D-Allose
Amygdalin
D-Arabinose
D-Arabitol
L-Arabitol
Arbutin
2-Deoxy-D-Ribose
i-Erythritol
D-Fucose
3-O-b-D-Galactopyranosyl-D-Arabinose
Gentiobiose
L-Glucose
D-Lactitol
D-Melezitose
Maltitol
a-Methyl-D-Glucoside
b-Methyl-D-Galactoside
3-Methylglucose
b-Methyl-D-Glucuronic acid
a-Methyl-D-Mannoside
b-Methyl-D-Xyloside
Palatinose
D-Raffinose
Salicin
Sedoheptulosan
L-Sorbose
Stachyose
D-Tagatose
Turanose
Xylitol
N-Acetyl-D-Glucosaminitol
g-Amino-N-Butyric acid
d-Amino Valeric acid
Butyric acid
Capric acid
Caproic acid
Citraconic acid
Citramalic acid
D-Glucosamine
2-Hydroxybenzoic acid
4-Hydroxybenzoic acid
b-Hydroxybutyric acid
g-Hydroxybutyric acid
a-Keto-Valeric acid
Itaconic acid
5-Keto-D-Gluconic acid
D-Lactic acid Methyl Ester
Malonic acid
Melibionic acid
Oxalic acid
Oxalomalic acid
Quinic acid
D-Ribono-1,4-Lactone
Sebacic acid
Sorbic acid
Succinamic acid
D-Tartaric acid
L-Tartaric acid
Acetamide
L-Alaninamide
N-Acetyl-L-Glutamic acid
L-Arginine
Glycine
L-Histidine
L-Homoserine
Hydroxy-L-Proline
L-Isoleucine
L-Leucine
L-Lysine
L-Methionine
L-Ornithine
L-Phenylalanine
L-Pyroglutamic acid
L-Valine
D,L-Carnitine
sec-Butylamine
D,L-Octopamine
Putrescine
Dihydroxyacetone
2,3-Butanediol
2,3-Butanedione
3-Hydroxy-2-buta
AR #0382
AR #0387
AR #0388
AR #0389
AR #0390
AR #0381
AR #0383
AR #0384
AR #0385
AR #0386
AR #0382
AR #0387
AR #0388
AR #0389
AR #0390
AR #0381
AR #0383
AR #0384
AR #0385
AR #0386
Exponential growth
yes
no
AUC
0
2000
4000
6000

C

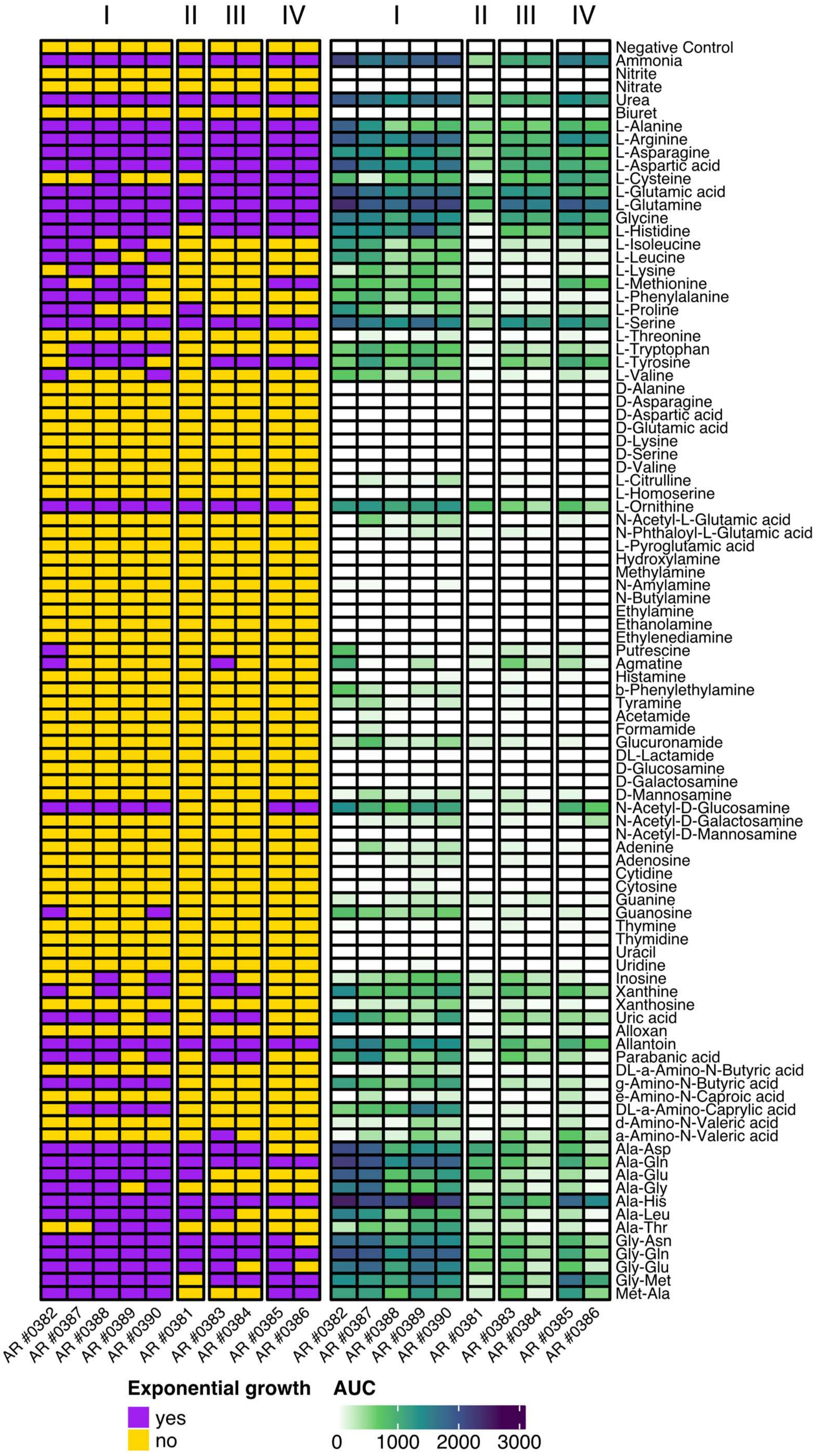

I
II
III
IV
I
II
III
IV
Negative Control
Ammonia
Nitrite
Nitrate
Urea
Biuret
L-Alanine
L-Arginine
L-Asparagine
L-Aspartic acid
L-Cysteine
L-Glutamic acid
L-Glutamine
Glycine
L-Histidine
L-Isoleucine
L-Leucine
L-Lysine
L-Methionine
L-Phenylalanine
L-Proline
L-Serine
L-Threonine
L-Tryptophan
L-Tyrosine
L-Valine
D-Alanine
D-Asparagine
D-Aspartic acid
D-Glutamic acid
D-Lysine
D-Serine
D-Valine
L-Citrulline
L-Homoserine
L-Ornithine
N-Acetyl-L-Glutamic acid
N-Phthaloyl-L-Glutamic acid
L-Pyroglutamic acid
Hydroxylamine
Methylamine
N-Amylamine
N-Butylamine
Ethylamine
Ethanolamine
Ethylenediamine
Putrescine
Agmatine
Histamine
b-Phenylethylamine
Tyramine
Acetamide
Formamide
Glucuronamide
DL-Lactamide
D-Glucosamine
D-Galactosamine
D-Mannosamine
N-Acetyl-D-Glucosamine
N-Acetyl-D-Galactosamine
N-Acetyl-D-Mannosamine
Adenine
Adenosine
Cytidine
Cytosine
Guanine
Guanosine
Thymine
Thymidine
Uracil
Uridine
Inosine
Xanthine
Xanthosine
Uric acid
Alloxan
Allantoin
Parabanic acid
DL-a-Amino-N-Butyric acid
g-Amino-N-Butyric acid
e-Amino-N-Caproic acid
DL-a-Amino-Caprylic acid
d-Amino-N-Valeric acid
a-Amino-N-Valeric acid
Ala-Asp
Ala-Gln
Ala-Glu
Ala-Gly
Ala-His
Ala-Leu
Ala-Thr
Gly-Asn
Gly-Gln
Gly-Glu
Gly-Met
Met-Ala
AR #0382
AR #0387
AR #0388
AR #0389
AR #0390
AR #0381
AR #0383
AR #0384
AR #0385
AR #0386
AR #0382
AR #0387
AR #0388
AR #0389
AR #0390
AR #0381
AR #0383
AR #0384
AR #0385
AR #0386
Exponential growth
yes
no
AUC
0
1000
2000
3000

D

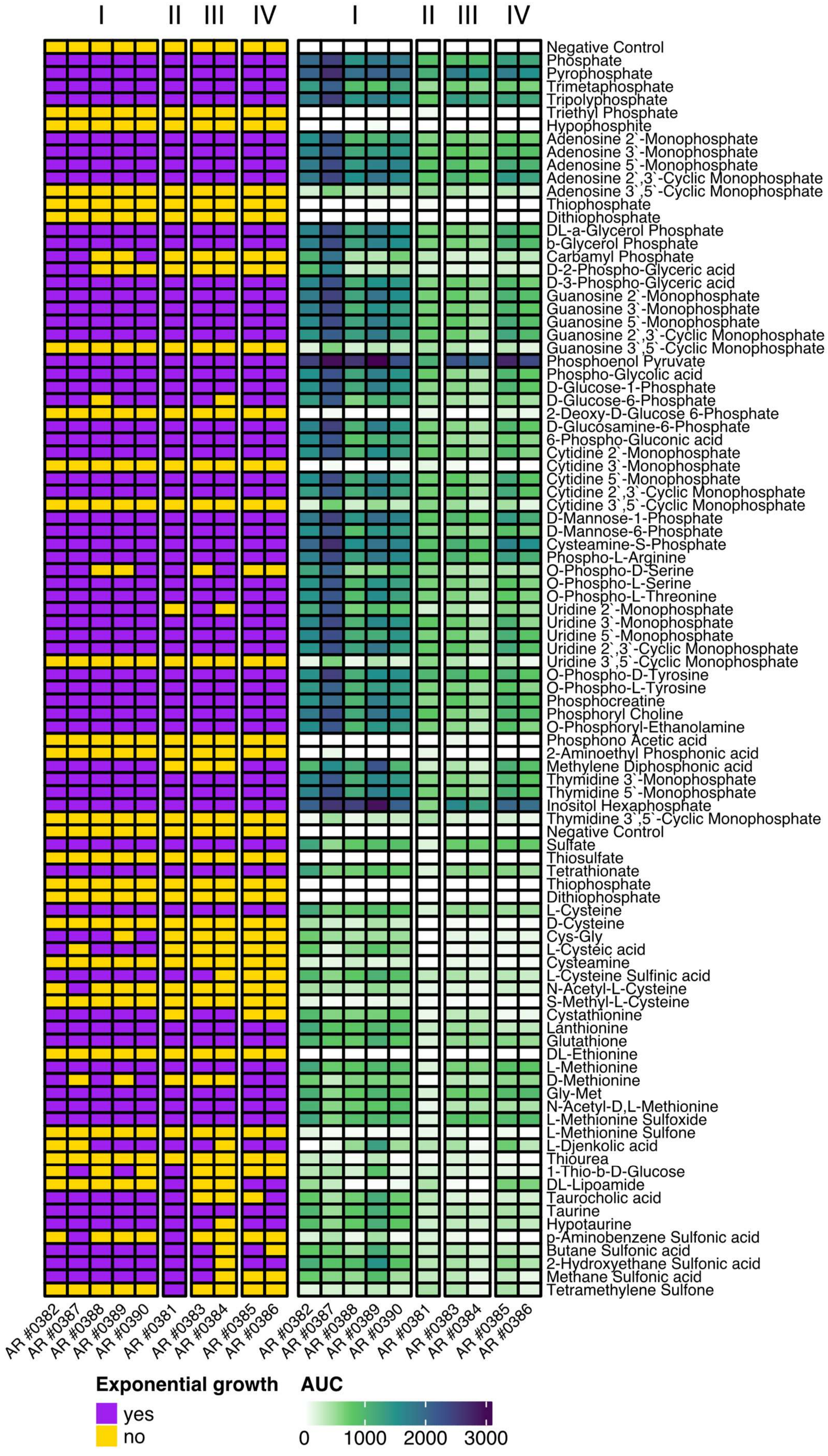

I
II
III
IV
Negative Control
Phosphate
Pyrophosphate
Trimetaphosphate
Tripolyphosphate
Triethyl Phosphate
Hypophosphite
Adenosine 2`-Monophosphate
Adenosine 3`-Monophosphate
Adenosine 5`-Monophosphate
Adenosine 2`,3`-Cyclic Monophosphate
Adenosine 3`,5`-Cyclic Monophosphate
Thiophosphate
Dithiophosphate
DL-a-Glycerol Phosphate
b-Glycerol Phosphate
Carbamyl Phosphate
D-2-Phospho-Glyceric acid
D-3-Phospho-Glyceric acid
Guanosine 2`-Monophosphate
Guanosine 3`-Monophosphate
Guanosine 5`-Monophosphate
Guanosine 2`,3`-Cyclic Monophosphate
Guanosine 3`,5`-Cyclic Monophosphate
Phosphoenol Pyruvate
Phospho-Glycolic acid
D-Glucose-1-Phosphate
D-Glucose-6-Phosphate
2-Deoxy-D-Glucose 6-Phosphate
D-Glucosamine-6-Phosphate
6-Phospho-Gluconic acid
Cytidine 2`-Monophosphate
Cytidine 3`-Monophosphate
Cytidine 5`-Monophosphate
Cytidine 2`,3`-Cyclic Monophosphate
Cytidine 3`,5`-Cyclic Monophosphate
D-Mannose-1-Phosphate
D-Mannose-6-Phosphate
Cysteamine-S-Phosphate
Phospho-L-Arginine
O-Phospho-D-Serine
O-Phospho-L-Serine
O-Phospho-L-Threonine
Uridine 2`-Monophosphate
Uridine 3`-Monophosphate
Uridine 5`-Monophosphate
Uridine 2`,3`-Cyclic Monophosphate
Uridine 3`,5`-Cyclic Monophosphate
O-Phospho-D-Tyrosine
O-Phospho-L-Tyrosine
Phosphocreatine
Phosphoryl Choline
O-Phosphoryl-Ethanolamine
Phosphono Acetic acid
2-Aminoethyl Phosphonic acid
Methylene Diphosphonic acid
Thymidine 3`-Monophosphate
Thymidine 5`-Monophosphate
Inositol Hexaphosphate
Thymidine 3`,5`-Cyclic Monophosphate
Negative Control
Sulfate
Thiosulfate
Tetrathionate
Thiophosphate
Dithiophosphate
L-Cysteine
D-Cysteine
Cys-Gly
L-Cysteic acid
Cysteamine
L-Cysteine Sulfinic acid
N-Acetyl-L-Cysteine
S-Methyl-L-Cysteine
Cystathionine
Lanthionine
Glutathione
DL-Ethionine
L-Methionine
D-Methionine
Gly-Met
N-Acetyl-D,L-Methionine
L-Methionine Sulfoxide
L-Methionine Sulfone
L-Djenkolic acid
Thiourea
1-Thio-b-D-Glucose
DL-Lipoamide
Taurocholic acid
Taurine
Hypotaurine
p-Aminobenzene Sulfonic acid
Butane Sulfonic acid
2-Hydroxyethane Sulfonic acid
Methane Sulfonic acid
Tetramethylene Sulfone
AR #0382
AR #0387
AR #0388
AR #0389
AR #0390
AR #0381
AR #0383
AR #0384
AR #0385
AR #0386
Exponential growth
yes
no
AUC
0
1000
2000
3000

E

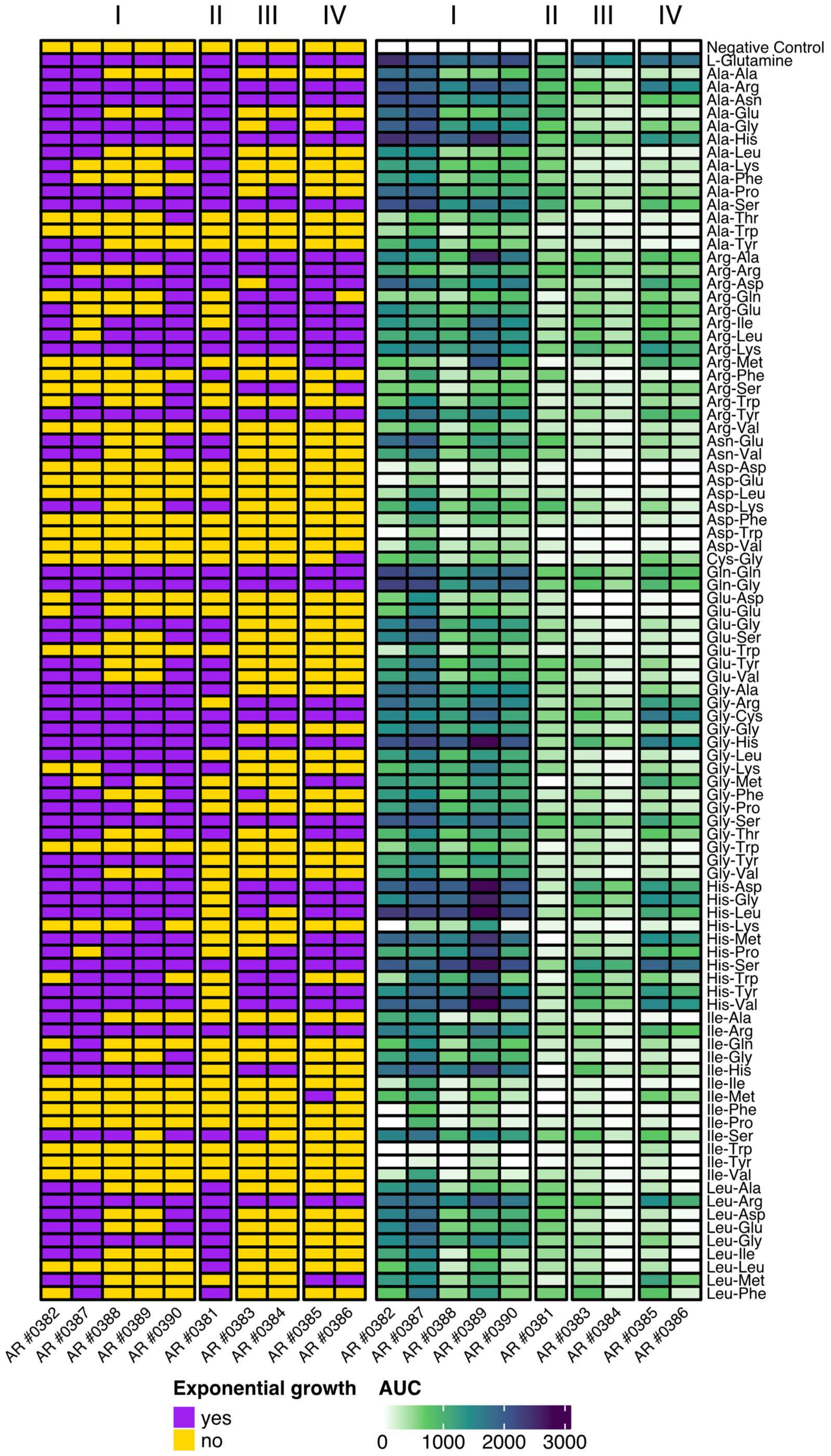

I
II
III
IV
I
II
III
IV
Negative Control
L-Glutamine
Ala-Ala
Ala-Arg
Ala-Asn
Ala-Glu
Ala-Gly
Ala-His
Ala-Leu
Ala-Lys
Ala-Phe
Ala-Pro
Ala-Ser
Ala-Thr
Ala-Trp
Ala-Tyr
Arg-Ala
Arg-Arg
Arg-Asp
Arg-Gln
Arg-Glu
Arg-Ile
Arg-Leu
Arg-Lys
Arg-Met
Arg-Phe
Arg-Ser
Arg-Trp
Arg-Tyr
Arg-Val
Asn-Glu
Asn-Val
Asp-Asp
Asp-Glu
Asp-Leu
Asp-Lys
Asp-Phe
Asp-Trp
Asp-Val
Cys-Gly
Gln-Gln
Gln-Gly
Glu-Asp
Glu-Glu
Glu-Gly
Glu-Ser
Glu-Trp
Glu-Tyr
Glu-Val
Gly-Ala
Gly-Arg
Gly-Cys
Gly-Gly
Gly-His
Gly-Leu
Gly-Lys
Gly-Met
Gly-Phe
Gly-Pro
Gly-Ser
Gly-Thr
Gly-Trp
Gly-Tyr
Gly-Val
His-Asp
His-Gly
His-Leu
His-Lys
His-Met
His-Pro
His-Ser
His-Trp
His-Tyr
His-Val
Ile-Ala
Ile-Arg
Ile-Gln
Ile-Gly
Ile-His
Ile-Ile
Ile-Met
Ile-Phe
Ile-Pro
Ile-Ser
Ile-Trp
Ile-Tyr
Ile-Val
Leu-Ala
Leu-Arg
Leu-Asp
Leu-Glu
Leu-Gly
Leu-Ile
Leu-Leu
Leu-Met
Leu-Phe
AR #0382
AR #0387
AR #0388
AR #0389
AR #0390
AR #0381
AR #0383
AR #0384
AR #0385
AR #0386
AR #0382
AR #0387
AR #0388
AR #0389
AR #0390
AR #0381
AR #0383
AR #0384
AR #0385
AR #0386
Exponential growth
yes
no
AUC
0
1000
2000
3000

F

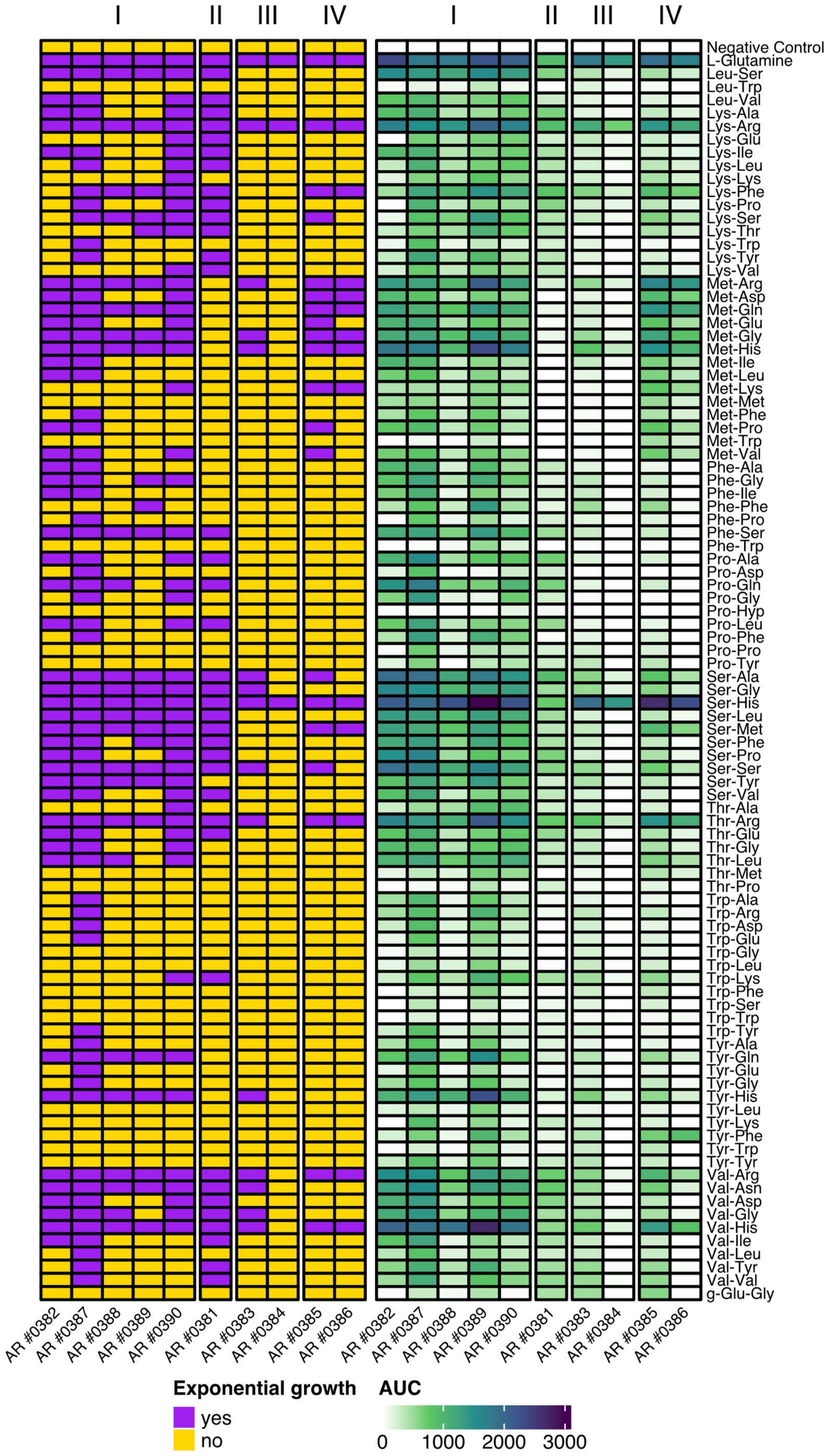
I
II
III
IV
Negative Control
L-Glutamine
Leu-Ser
Leu-Trp
Leu-Val
Lys-Ala
Lys-Arg
Lys-Glu
Lys-Ile
Lys-Leu
Lys-Lys
Lys-Phe
Lys-Pro
Lys-Ser
Lys-Thr
Lys-Trp
Lys-Tyr
Lys-Val
Met-Arg
Met-Asp
Met-Gln
Met-Glu
Met-Gly
Met-His
Met-Ile
Met-Leu
Met-Lys
Met-Met
Met-Phe
Met-Pro
Met-Trp
Met-Val
Phe-Ala
Phe-Gly
Phe-Ile
Phe-Phe
Phe-Pro
Phe-Ser
Phe-Trp
Pro-Ala
Pro-Asp
Pro-Gln
Pro-Gly
Pro-Hyp
Pro-Leu
Pro-Phe
Pro-Pro
Pro-Tyr
Ser-Ala
Ser-Gly
Ser-His
Ser-Leu
Ser-Met
Ser-Phe
Ser-Pro
Ser-Ser
Ser-Tyr
Ser-Val
Thr-Ala
Thr-Arg
Thr-Glu
Thr-Gly
Thr-Leu
Thr-Met
Thr-Pro
Trp-Ala
Trp-Arg
Trp-Asp
Trp-Glu
Trp-Gly
Trp-Leu
Trp-Lys
Trp-Phe
Trp-Ser
Trp-Trp
Trp-Tyr
Tyr-Ala
Tyr-Gln
Tyr-Glu
Tyr-Gly
Tyr-His
Tyr-Leu
Tyr-Lys
Tyr-Phe
Tyr-Trp
Tyr-Tyr
Val-Arg
Val-Asn
Val-Asp
Val-Gly
Val-His
Val-Ile
Val-Leu
Val-Tyr
Val-Val
g-Glu-Gly
AR #0382
AR #0387
AR #0388
AR #0389
AR #0390
AR #0381
AR #0383
AR #0384
AR #0385
AR #0386
Exponential growth
yes
no
AUC
0
1000
2000
3000

G

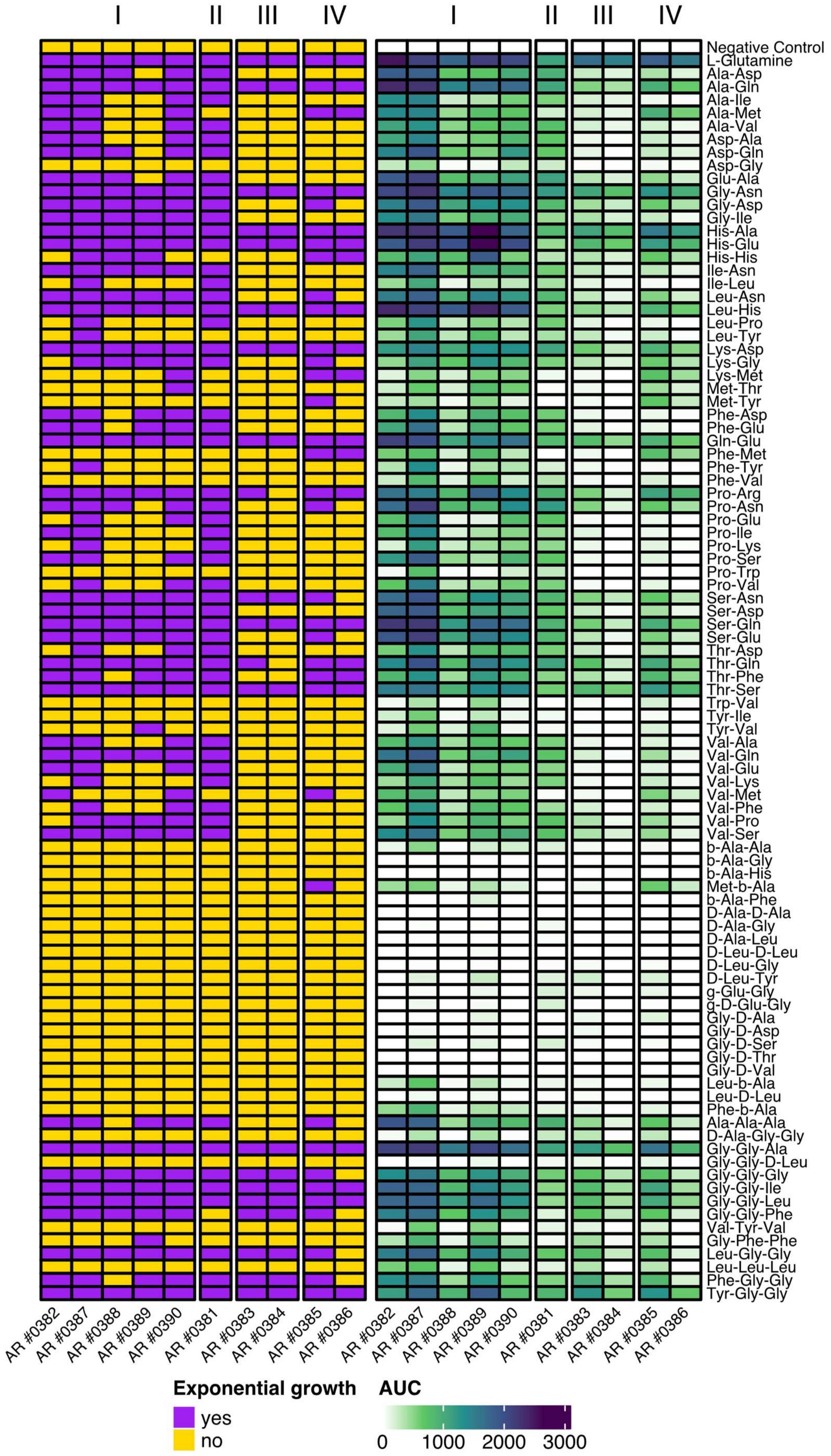

I
II
III
IV
I
II
III
IV
Negative Control
L-Glutamine
Ala-Asp
Ala-Gln
Ala-Ile
Ala-Met
Ala-Val
Asp-Ala
Asp-Gln
Asp-Gly
Glu-Ala
Gly-Asn
Gly-Asp
Gly-Ile
His-Ala
His-Glu
His-His
Ile-Asn
Ile-Leu
Leu-Asn
Leu-His
Leu-Pro
Leu-Tyr
Lys-Asp
Lys-Gly
Lys-Met
Met-Thr
Met-Tyr
Phe-Asp
Phe-Glu
Gln-Glu
Phe-Met
Phe-Tyr
Phe-Val
Pro-Arg
Pro-Asn
Pro-Glu
Pro-Ile
Pro-Lys
Pro-Ser
Pro-Trp
Pro-Val
Ser-Asn
Ser-Asp
Ser-Gln
Ser-Glu
Thr-Asp
Thr-Gln
Thr-Phe
Thr-Ser
Trp-Val
Tyr-Ile
Tyr-Val
Val-Ala
Val-Gln
Val-Glu
Val-Lys
Val-Met
Val-Phe
Val-Pro
Val-Ser
b-Ala-Ala
b-Ala-Gly
b-Ala-His
Met-b-Ala
b-Ala-Phe
D-Ala-D-Ala
D-Ala-Gly
D-Ala-Leu
D-Leu-D-Leu
D-Leu-Gly
D-Leu-Tyr
g-Glu-Gly
g-D-Glu-Gly
Gly-D-Ala
Gly-D-Asp
Gly-D-Ser
Gly-D-Thr
Gly-D-Val
Leu-b-Ala
Leu-D-Leu
Phe-b-Ala
Ala-Ala-Ala
D-Ala-Gly-Gly
Gly-Gly-Ala
Gly-Gly-D-Leu
Gly-Gly-Gly
Gly-Gly-Ile
Gly-Gly-Leu
Gly-Gly-Phe
Val-Tyr-Val
Gly-Phe-Phe
Leu-Gly-Gly
Leu-Leu-Leu
Phe-Gly-Gly
Tyr-Gly-Gly
AR #0382
AR #0387
AR #0388
AR #0389
AR #0390
AR #0381
AR #0383
AR #0384
AR #0385
AR #0386
AR #0382
AR #0387
AR #0388
AR #0389
AR #0390
AR #0381
AR #0383
AR #0384
AR #0385
AR #0386
Exponential growth
yes
no
AUC
0
1000
2000
3000

H

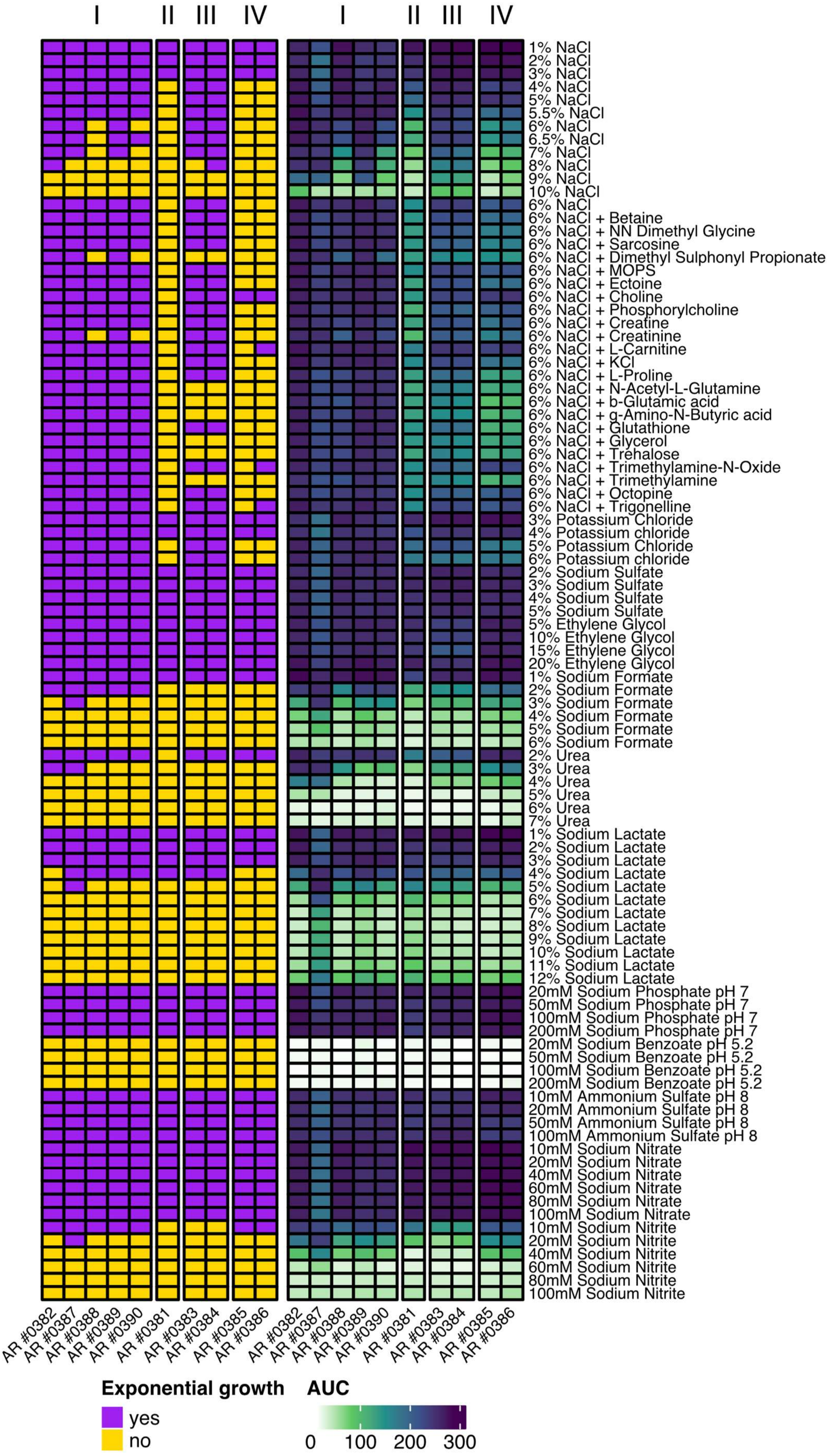
I
II
III
IV
1% NaCl
2% NaCl
3% NaCl
4% NaCl
5% NaCl
5.5% NaCl
6% NaCl
6.5% NaCl
7% NaCl
8% NaCl
9% NaCl
10% NaCl
6% NaCl
6% NaCl + Betaine
6% NaCl + NN Dimethyl Glycine
6% NaCl + Sarcosine
6% NaCl + Dimethyl Sulphonyl Propionate
6% NaCl + MOPS
6% NaCl + Ectoine
6% NaCl + Choline
6% NaCl + Phosphorylcholine
6% NaCl + Creatine
6% NaCl + Creatinine
6% NaCl + L-Carnitine
6% NaCl + KCl
6% NaCl + L-Proline
6% NaCl + N-Acetyl-L-Glutamine
6% NaCl + b-Glutamic acid
6% NaCl + g-Amino-N-Butyric acid
6% NaCl + Glutathione
6% NaCl + Glycerol
6% NaCl + Trehalose
6% NaCl + Trimethylamine-N-Oxide
6% NaCl + Trimethylamine
6% NaCl + Octopine
6% NaCl + Trigonelline
3% Potassium Chloride
4% Potassium chloride
5% Potassium Chloride
6% Potassium chloride
2% Sodium Sulfate
3% Sodium Sulfate
4% Sodium Sulfate
5% Sodium Sulfate
5% Ethylene Glycol
10% Ethylene Glycol
15% Ethylene Glycol
20% Ethylene Glycol
1% Sodium Formate
2% Sodium Formate
3% Sodium Formate
4% Sodium Formate
5% Sodium Formate
6% Sodium Formate
2% Urea
3% Urea
4% Urea
5% Urea
6% Urea
7% Urea
1% Sodium Lactate
2% Sodium Lactate
3% Sodium Lactate
4% Sodium Lactate
5% Sodium Lactate
6% Sodium Lactate
7% Sodium Lactate
8% Sodium Lactate
9% Sodium Lactate
10% Sodium Lactate
11% Sodium Lactate
12% Sodium Lactate
20mM Sodium Phosphate pH 7
50mM Sodium Phosphate pH 7
100mM Sodium Phosphate pH 7
200mM Sodium Phosphate pH 7
20mM Sodium Benzoate pH 5.2
50mM Sodium Benzoate pH 5.2
100mM Sodium Benzoate pH 5.2
200mM Sodium Benzoate pH 5.2
10mM Ammonium Sulfate pH 8
20mM Ammonium Sulfate pH 8
50mM Ammonium Sulfate pH 8
100mM Ammonium Sulfate pH 8
10mM Sodium Nitrate
20mM Sodium Nitrate
40mM Sodium Nitrate
60mM Sodium Nitrate
80mM Sodium Nitrate
100mM Sodium Nitrate
10mM Sodium Nitrite
20mM Sodium Nitrite
40mM Sodium Nitrite
60mM Sodium Nitrite
80mM Sodium Nitrite
100mM Sodium Nitrite
AR #0382
AR #0387
AR #0388
AR #0389
AR #0390
AR #0381
AR #0383
AR #0384
AR #0385
AR #0386
Exponential growth
yes
no
AUC
0
100
200
300

I

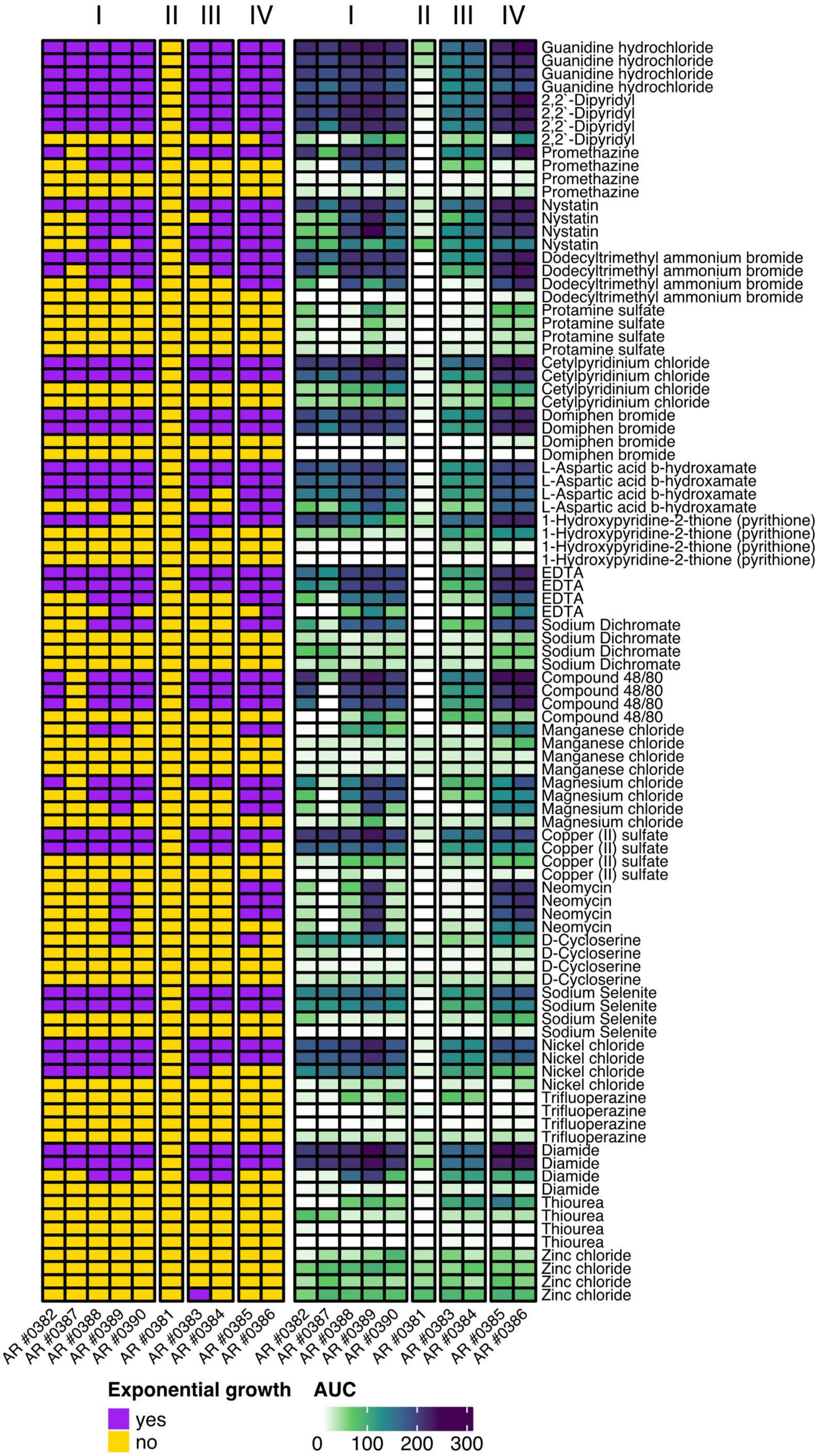

I
II
III
IV
I
II
III
IV
Guanidine hydrochloride
Guanidine hydrochloride
Guanidine hydrochloride
Guanidine hydrochloride
2,2`-Dipyridyl
2,2`-Dipyridyl
2,2`-Dipyridyl
2,2`-Dipyridyl
Promethazine
Promethazine
Promethazine
Promethazine
Nystatin
Nystatin
Nystatin
Nystatin
Dodecyltrimethyl ammonium bromide
Dodecyltrimethyl ammonium bromide
Dodecyltrimethyl ammonium bromide
Dodecyltrimethyl ammonium bromide
Protamine sulfate
Protamine sulfate
Protamine sulfate
Protamine sulfate
Cetylpyridinium chloride
Cetylpyridinium chloride
Cetylpyridinium chloride
Cetylpyridinium chloride
Domiphen bromide
Domiphen bromide
Domiphen bromide
Domiphen bromide
L-Aspartic acid b-hydroxamate
L-Aspartic acid b-hydroxamate
L-Aspartic acid b-hydroxamate
L-Aspartic acid b-hydroxamate
1-Hydroxypyridine-2-thione (pyrithione)
1-Hydroxypyridine-2-thione (pyrithione)
1-Hydroxypyridine-2-thione (pyrithione)
1-Hydroxypyridine-2-thione (pyrithione)
EDTA
EDTA
EDTA
EDTA
Sodium Dichromate
Sodium Dichromate
Sodium Dichromate
Sodium Dichromate
Compound 48/80
Compound 48/80
Compound 48/80
Compound 48/80
Manganese chloride
Manganese chloride
Manganese chloride
Manganese chloride
Magnesium chloride
Magnesium chloride
Magnesium chloride
Magnesium chloride
Copper (II) sulfate
Copper (II) sulfate
Copper (II) sulfate
Copper (II) sulfate
Neomycin
Neomycin
Neomycin
Neomycin
D-Cycloserine
D-Cycloserine
D-Cycloserine
D-Cycloserine
Sodium Selenite
Sodium Selenite
Sodium Selenite
Sodium Selenite
Nickel chloride
Nickel chloride
Nickel chloride
Nickel chloride
Trifluoperazine
Trifluoperazine
Trifluoperazine
Trifluoperazine
Diamide
Diamide
Diamide
Diamide
Thiourea
Thiourea
Thiourea
Thiourea
Zinc chloride
Zinc chloride
Zinc chloride
Zinc chloride
AR #0382
AR #0387
AR #0388
AR #0389
AR #0390
AR #0381
AR #0383
AR #0384
AR #0385
AR #0386
AR #0382
AR #0387
AR #0388
AR #0389
AR #0390
AR #0381
AR #0383
AR #0384
AR #0385
AR #0386
Exponential growth
yes
no
AUC
0
100
200
300

J

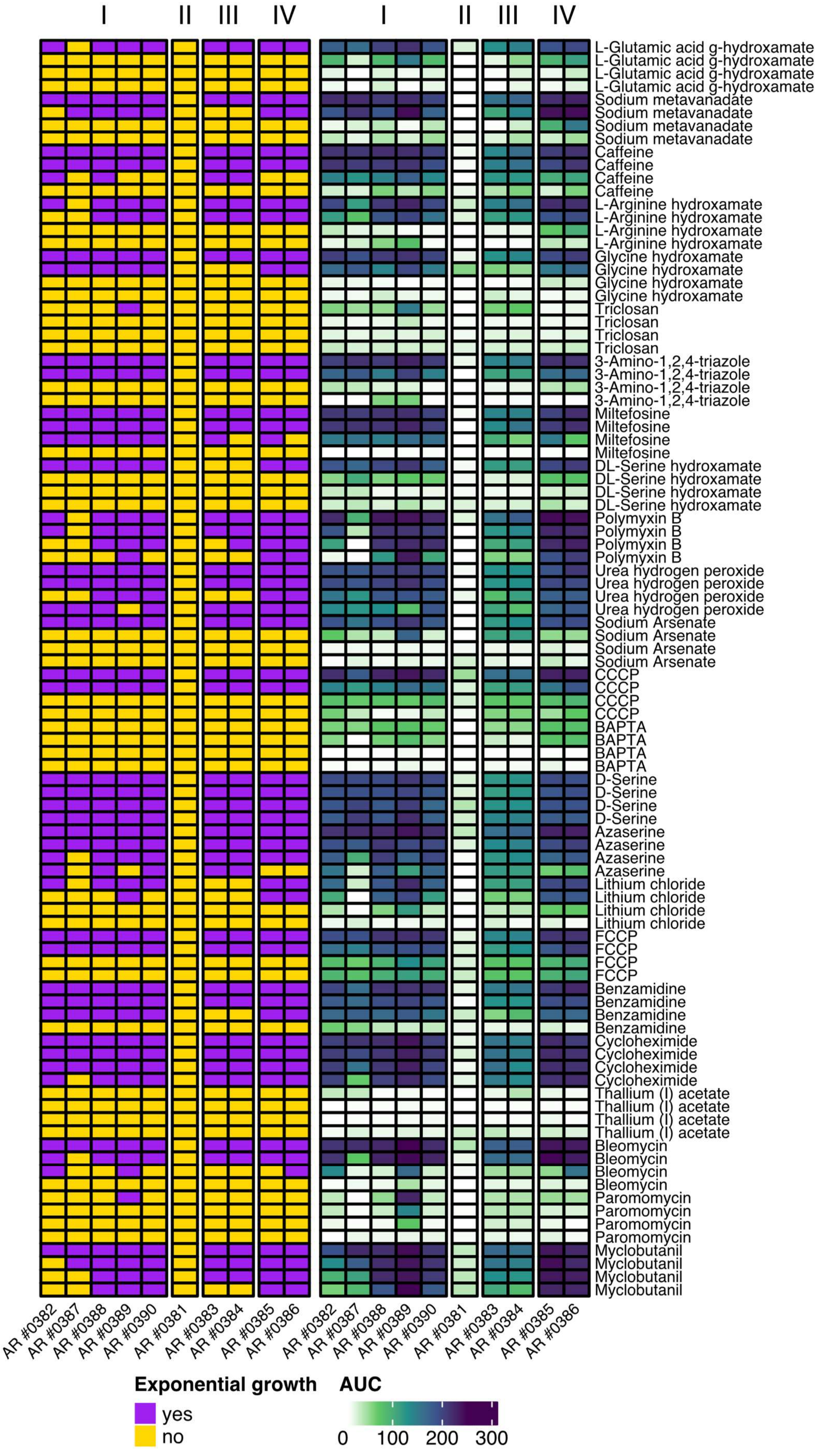
I
II
III
IV
L-Glutamic acid g-hydroxamate
Sodium metavanadate
Caffeine
L-Arginine hydroxamate
Glycine hydroxamate
Triclosan
3-Amino-1,2,4-triazole
Miltefosine
DL-Serine hydroxamate
Polymyxin B
Urea hydrogen peroxide
Sodium Arsenate
CCCP
BAPTA
D-Serine
Azaserine
Lithium chloride
FCCP
Benzamidine
Cycloheximide
Thallium (I) acetate
Bleomycin
Paromomycin
Myclobutanil
AR #0382
AR #0387
AR #0388
AR #0389
AR #0390
AR #0381
AR #0383
AR #0384
AR #0385
AR #0386
Exponential growth
yes
no
AUC
0
100
200
300

K

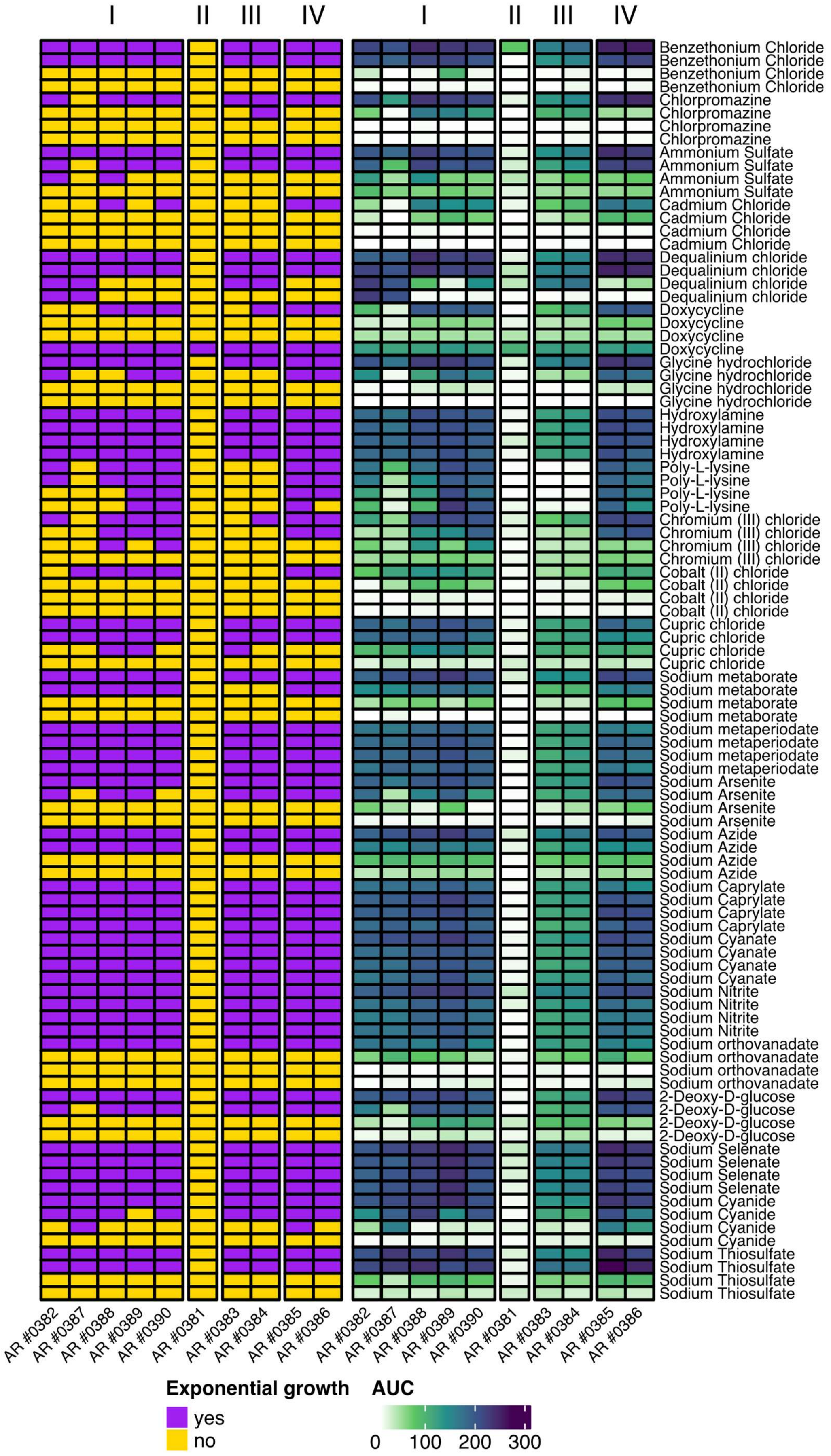
I
II
III
IV
Benzethonium Chloride
Chlorpromazine
Ammonium Sulfate
Cadmium Chloride
Dequalinium chloride
Doxycycline
Glycine hydrochloride
Hydroxylamine
Poly-L-lysine
Chromium (III) chloride
Cobalt (II) chloride
Cupric chloride
Sodium metaborate
Sodium metaperiodate
Sodium Arsenite
Sodium Azide
Sodium Caprylate
Sodium Cyanate
Sodium Nitrite
Sodium orthovanadate
2-Deoxy-D-glucose
Sodium Selenate
Sodium Cyanide
Sodium Thiosulfate
AR #0382
AR #0387
AR #0388
AR #0389
AR #0390
AR #0381
AR #0383
AR #0384
AR #0385
AR #0386
Exponential growth
yes
no
AUC
0
100
200
300

L

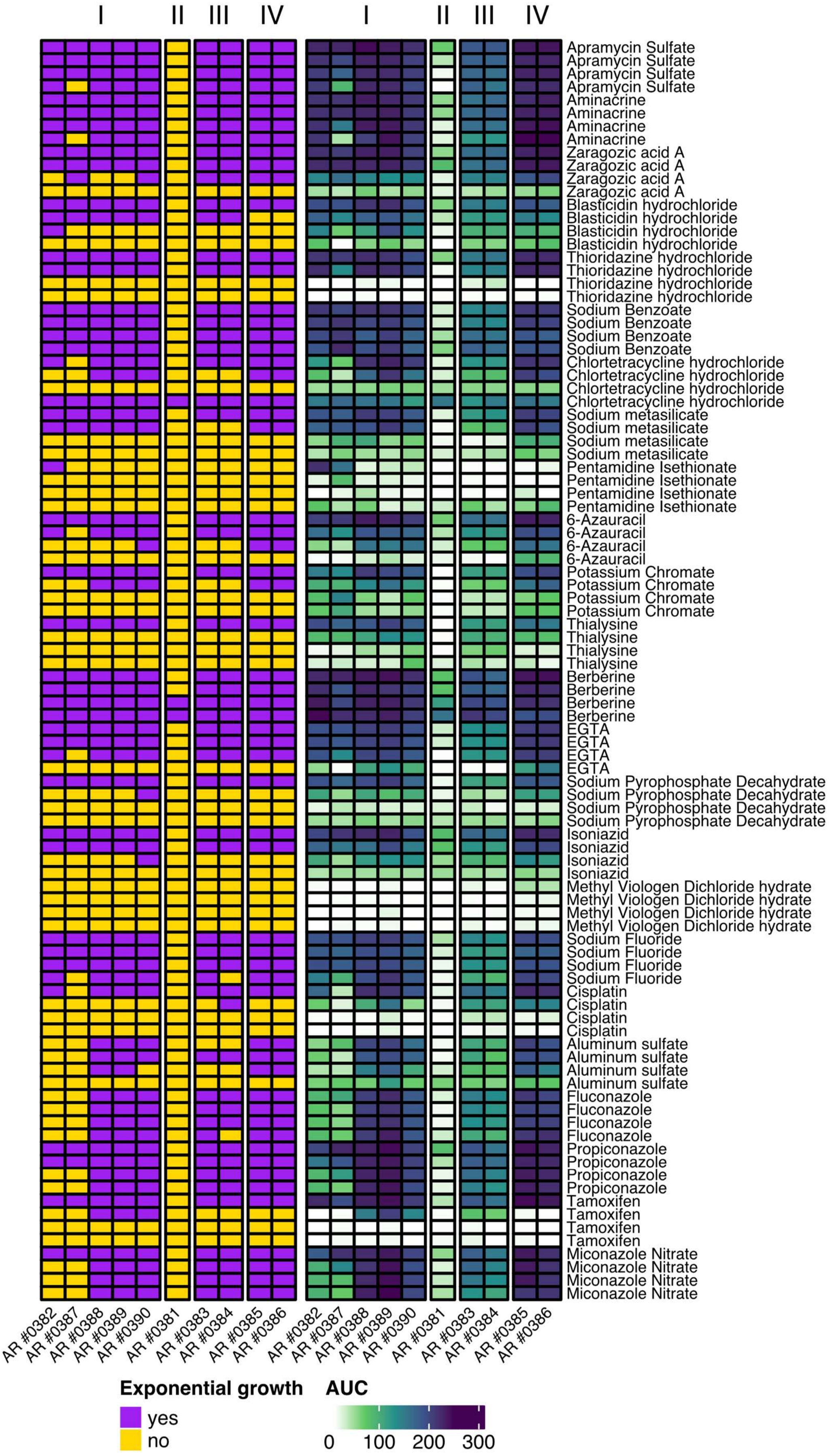

I
II
III
IV
I
II
III
IV
Apramycin Sulfate
Aminacrine
Zaragozic acid A
Blasticidin hydrochloride
Thioridazine hydrochloride
Sodium Benzoate
Chlortetracycline hydrochloride
Sodium metasilicate
Pentamidine Isethionate
6-Azauracil
Potassium Chromate
Thialysine
Berberine
EGTA
Sodium Pyrophosphate Decahydrate
Isoniazid
Methyl Viologen Dichloride hydrate
Sodium Fluoride
Cisplatin
Aluminum sulfate
Fluconazole
Propiconazole
Tamoxifen
Miconazole Nitrate
AR #0382
AR #0387
AR #0388
AR #0389
AR #0390
AR #0381
AR #0383
AR #0384
AR #0385
AR #0386
Exponential growth
yes
no
AUC
0
100
200
300

M

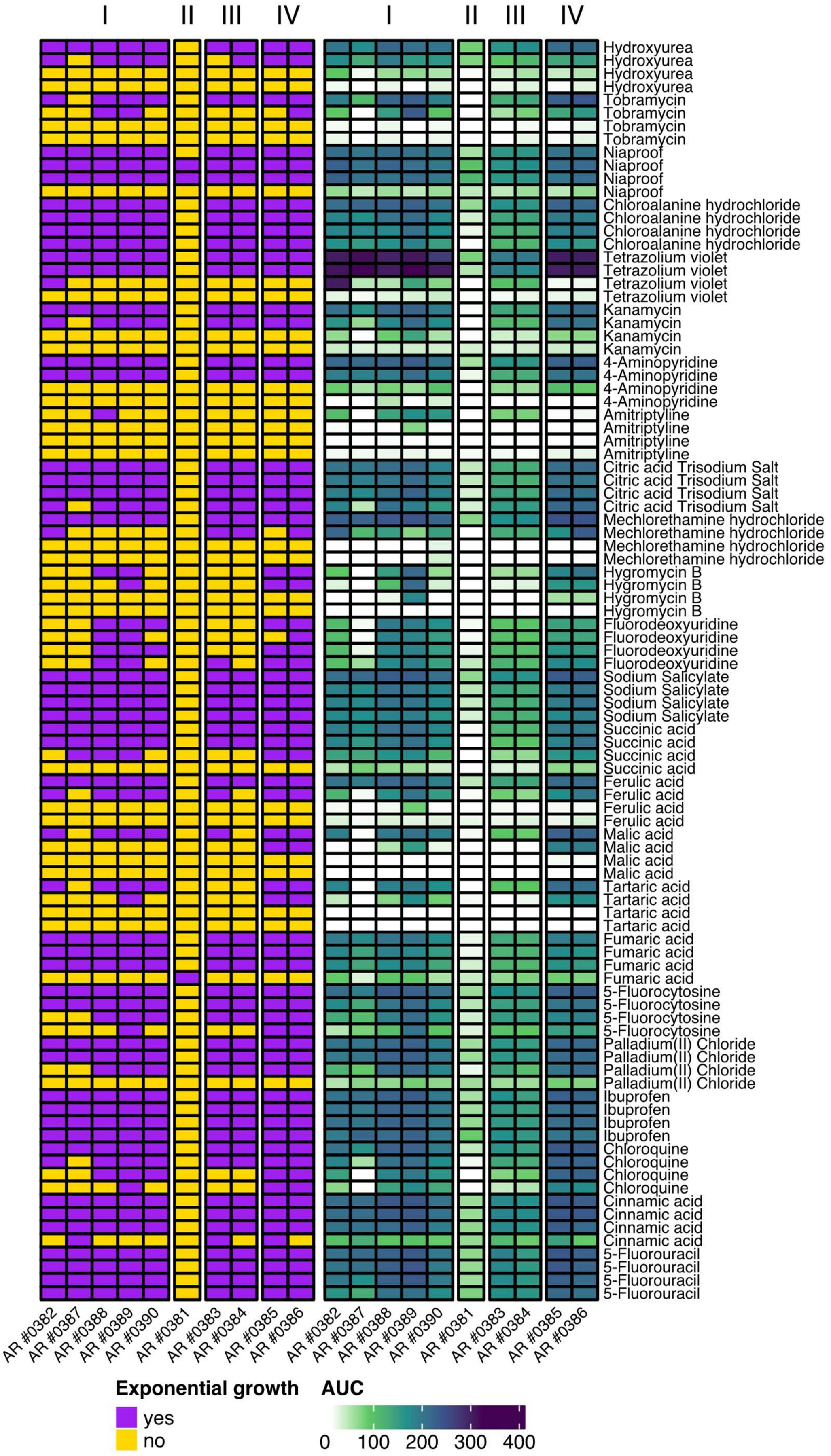

I
II
III
IV
Hydroxyurea
Tobramycin
Niaproof
Chloroalanine hydrochloride
Tetrazolium violet
Kanamycin
4-Aminopyridine
Amitriptyline
Citric acid Trisodium Salt
Mechlorethamine hydrochloride
Hygromycin B
Fluorodeoxyuridine
Sodium Salicylate
Succinic acid
Ferulic acid
Malic acid
Tartaric acid
Fumaric acid
5-Fluorocytosine
Palladium(II) Chloride
Ibuprofen
Chloroquine
Cinnamic acid
5-Fluorouracil
AR #0382
AR #0387
AR #0388
AR #0389
AR #0390
AR #0381
AR #0383
AR #0384
AR #0385
AR #0386
Exponential growth
yes
no
AUC
0
100
200
300
400

Figure S1: Biolog Phenotype MicroArray results.

Biolog Phenotype MicroArray plates for fungi were used to measure the metabolic activity of *C. auris* isolates kinetically every 15 min for 24 h or 48 h at 37°C, n = 3. **(A)** PM1 – carbon sources, **(B)** PM2 – carbon sources, **(C)** PM3 – nitrogen sources, **(D)** PM4 – phosphorus and sulfur sources, **(E)** PM6 – peptide nitrogen sources, **(F)** PM7 – peptide nitrogen sources, **(G)** PM8 – peptide nitrogen sources, **(H)** PM9 – osmolytes, **(I)** PM21 – chemicals, **(J)** PM22 – chemicals, **(K)** PM23 – chemicals, **(L)** PM24 – chemicals, **(M)** PM25 – chemicals. Left panel: Exponential growth phase according to the fitted logistic function of three biological replicates per strain. Right panel: Total growth over 24 h or 48 h calculated from the area under the curve (AUC).
